# Using online social networks to provide a parental health-education intervention for preventing unintentional injuries among children aged 0–3 years: A randomized controlled trial and social network analysis in Shanghai, China

**DOI:** 10.3389/fpubh.2022.1049851

**Published:** 2023-01-11

**Authors:** Yuheng Feng, Xiaohong Li, Xueqi Ma, Zhixu Zhu, Kaiyue Chen, Jun Gao, Jingwei Xia, Ruo Jiang, Jun Lu

**Affiliations:** ^1^Department of Health Policy and Management, School of Public Health, Fudan University, Shanghai, China; ^2^China Research Center on Disability Issues, Fudan University, Shanghai, China; ^3^Key Laboratory of Health Technology Assessment, National Health Commission, Fudan University, Shanghai, China; ^4^Informatization Office, Fudan University, Shanghai, China; ^5^Shanghai Huangpu District Maternal and Child Health Care Institute, Shanghai, China

**Keywords:** unintentional injury, social network analysis, WeChat, online social networks, randomized controlled trial

## Abstract

**Introduction:**

Unintentional injury among children represents a major public health problem. Online-social-network-based parental-health-education is a potential way to reduce child unintentional injuries. The study aimed to explore the mechanisms by which online-social-network-based health education may reduce the unintentional injuries among children aged 0–3 years.

**Methods:**

We conducted a participant-blinded, randomized controlled, online-social-network-based health-education intervention study from March 2019 to February 2020 in Shanghai. We established four WeChat groups (two intervention groups and two control groups). For the intervention groups, a doctor's assistant regularly delivered information regarding unintentional injuries among children, and community childcare doctors answered parents' questions concerning their children's health, including unintentional injuries. Meanwhile, the control groups did not receive any information from the assistant. The study selected one intervention group and one control group and compared the ego network and whole network indicators to determine the differences between the intervention and control groups.

**Results:**

In the intervention and control groups, 64.5% and 31.9% of the members, respectively, engaged in communication, and 1,736 and 273 records, respectively, were obtained. Regarding ego network, the doctor showed the largest network in the intervention group, and the size of the intervention group's network was twice that of the control group; the number of ties in the intervention group was nine times that of the control group. Fourteen and four parents in the intervention and control group played an active role, respectively. Regarding centrality, all WeChat groups were loose and multiple centers existed. Regarding subgroup cohesion, the intervention group had 28 cliques with 27 members, and the control group had 4 cliques with 4 members. For structural hole, 23.7% and 7.5% members in the intervention and control group actively participated in interactions, respectively, having strong control and influence over other parents; 69.2% and 59.1% members in the intervention and control group, respectively, had values of < 1.000, showing that they had strong ability to cross-jump structural holes.

**Discussion:**

Online-social-networks-based health education interventions could enhance communication among parents, and between parents and community childcare doctors, and also shorten the social distance between them. Thus, online-social-network-based parental-health-education-intervention can be a feasible and generalizable means of preventing unintentional injuries among children.

## 1. Introduction

Unintentional injury is an important public-health problem, not only because it is the major cause of death among children, but also because it can result in disability and heavy economic burden (1–6). West et al. (7) reported that children aged 0–4 years show high mortality from unintentional injuries, with the highest mortality being among children under 1 year old. From the perspective of time-data analysis, although a gradual declining trend of mortality in unintentional injuries among children under 5 years of age was reported, however, effective measures remain necessary to further reduce unintentional injuries (8–10).

Young children, such as children under five years of age, are vulnerable to unintentional injury, and their safety is dependent on their caregivers' (e.g., parents, grandparents, and babysitters) supervision (11–14). Thus, children's caregivers play an important role in protecting children from unintentional injuries. The main reasons for these children's vulnerability to unintentional injury include parents' lack of safety knowledge, parents' attitudes and behaviors, and the children's own attributes, such as impulsive and highly active (6, 12–16). Thus, it is essential to improve caregivers' knowledge, attitudes, and behaviors in this regard. Notably, studies have shown that health-education interventions for parents are an effective means of reducing unintentional injuries among children (17, 18).

The number of online social media users increase rapidly, such as Facebook and Twitter (19). In America, up to 46% people stated that they use social media as a sourcetool to look news in 2016, which is almost twice for 2013 (20). In China, mobile instant messaging users were up to 668 million in 2017 which covered 92.3% Chinese internet users. WeChat users, a popular mobile social app in China, covered 79.6% Chinese internet users (21, 22). As of 2019, the users' number of WeChat had reached about 1.17 billion, which was an increasing figure (23). So, interventions that are conducted through online social media can be considered promising. Additionally, this way also can be considered cost-effective (24). Notably, app-based interventions are widely used in health education (25–28).

Some apps, such as aforementioned software, are free to use and have large numbers of users. These popular apps, as convenient group-communication platforms, feature functions that are familiar to many people; for example, the facility to establish groups among people who have similar interests and goals. This suggests that such apps are suitable hosts for generalizable health-education interventions. Such apps can not only represent tools for delivering information, but also platforms for interaction. The effectiveness of online-social-network-based interventions has been confirmed in several areas of public health, such as mental health (29), smoking-prevention (30), and weight loss (31). Thus, online-social-network-based health-education interventions for parents represent a potential means of reducing the incidence of unintentional injuries among children. However, few studies have examined in detail the effect mechanism of online-social-network-based interventions (31); in addition, little is known about the mechanism underlying interactions among parents and health providers on online social media.

A small number of studies have explored app-based interventions for unintentional injuries among children (1). In a cluster randomized trial conducted in China, a new specialized app was developed to deliver to parents information concerning unintentional-injury-prevention and provide an interaction platform for parents and health-care providers; this study found that an app-based intervention can improve parents' knowledge and behaviors (32). However, its withdraw rate was higher than expected and developing a new app is expensive, which limits its generalizability. Therefore, there are several questions to be answered: (1) Can popular free apps be used as health education tools to prevent unintentional injuries among children aged 0–3 years? (2) What is the effect mechanism of such apps?

To investigate these questions, we previously conducted a randomized trial using WeChat, a free app that is very popular in China (33). Due to home is the main live environment for children aged 0–3 whose safety much more depends on their parents and seldom focused on them (1, 13, 14), the trial targeted parents of children aged 0–3 years, and confirmed the positive effect of a parental-health-education intervention based on online social media on reducing the occurrence of unintentional injuries *via* enhancing parents' associated knowledge, skills, and behaviors. In this study, we analyze the mechanism of interaction to understand the how such an online social network intervention can have this effect.

Social-network analysis (SNA) is a major social-science methodology that is used to analyze characteristics of social relationships; for example, sharing of values, capital, and knowledge among social units such as individuals, groups, and societies (34–36). In the health-care field, SNA has been widely used to characterize aspects such as relationships (37, 38), chronic disease management (39) and infectious disease (40); however, there has been little SNA-based investigation in relation to health education (41). A scoping review stated that SNA can not only be used to explore the function of complex interventions across different phases (42), but that SNA can graphically illustrate hidden relations between group members in social networks (43–45). Specifically, the main strength of SNA is its ability to allow researchers to examine mechanisms of interaction in online social networks (41).

In the present study, an online parental-health-education intervention was conducted through the use of WeChat groups that included a community childcare doctor, the doctor's assistant, and the target parents. SNA was used to analyze the mechanisms of health education *via* online social networks.

A characteristic element of the relationship among WeChat group members is information asymmetry, because community childcare doctors, as health-care providers, possess more knowledge about childcare, and parents, as healthcare demanders, lack such knowledge despite having the responsibility to care for their children. Hence, for the present study we created the following hypotheses:

*Research hypothesis H*_01_*: In WeChat groups, the community childcare doctor is situated at the core. In addition, parents who have better knowledge about child health and are ready to help others voluntarily answer other parents' questions*.
*Research hypothesis H*_02_*: The WeChat-group-based parental-health-education intervention facilitated by the doctor and the assistant encourages more parents to participate in the interactions between group members*.
*Research hypothesis H*_03_*: The WeChat-group-based parental-health-education intervention can connect parents and promote their acquiring of childcare knowledge via mutual communication with other parents*.

## 2. Materials and methods

### 2.1. Study design

**Figure 1:**
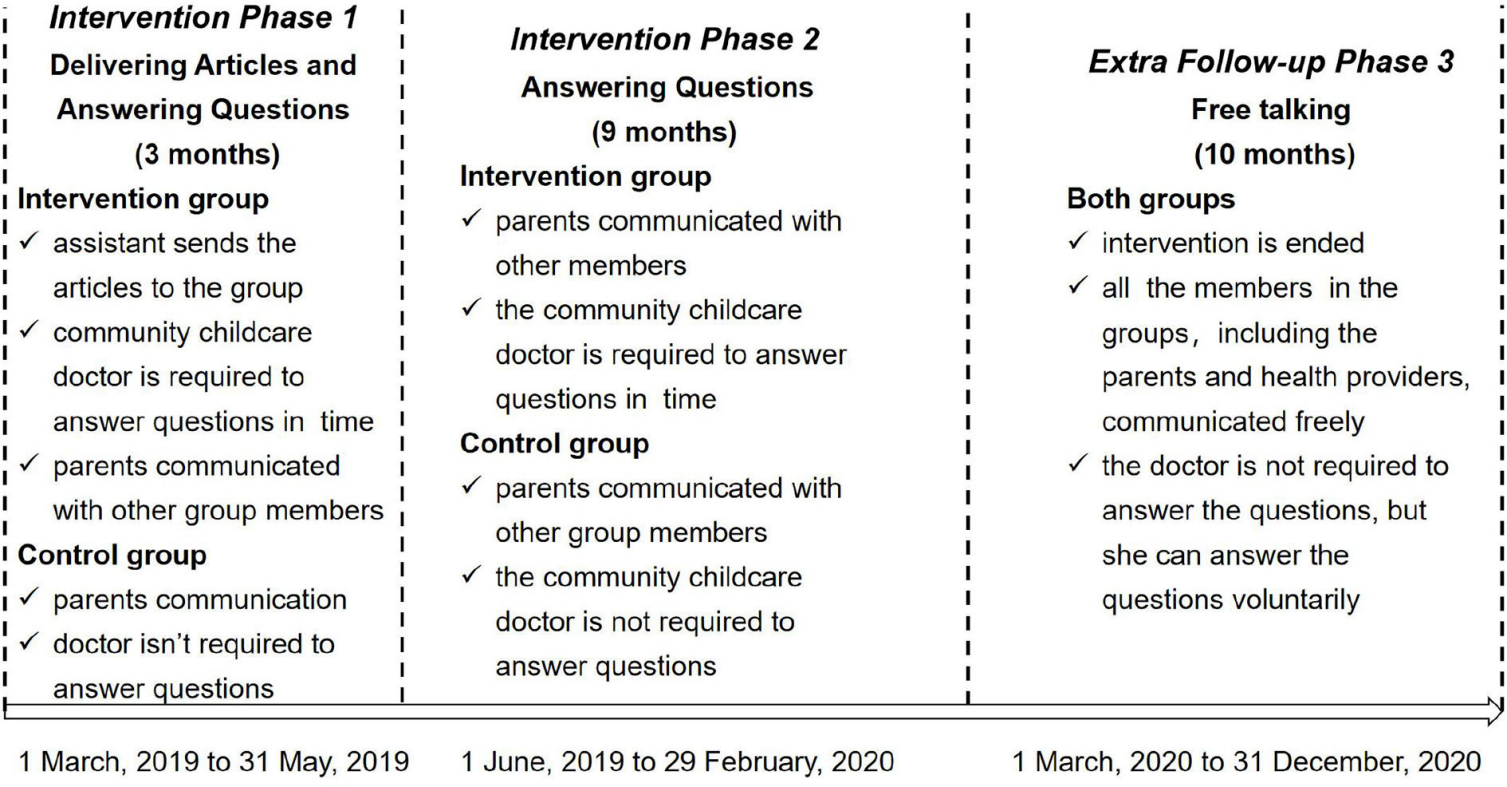
The phases of study design.

The study was conducted in the central area of Jiading District, Shanghai, China, from March 2019 to December 2020, and featured three phases (Figure 1).

Phase 1 comprised a 12-week (~3 months) health-education intervention designed to improve parents' knowledge, skills, and behaviors regarding unintentional injuries among children. The Phase 1 process was conducted by the assistant to the community childcare doctor, who posted 2–3 articles per week on the official WeChat account and also sent the articles to the intervention groups (also *via* WeChat), reminding parents to read them. The parents could also ask questions and communicate with each other during this period. The doctor was required to answer parents' questions within 48 h.

Phase 2 comprised the 9 months following Phase 1, during which parents could continue to communicate with each other and ask the community childcare doctor questions about childcare. The difference between the intervention and control group during this phase was that the community childcare doctor was not required to answer the control-group questions within 48 h.

Phase 3 comprised an additional follow-up, which lasted 10 months; this represented the post-intervention period, and provided an indication of the group members' voluntary behaviors regarding seeking and providing information concerning unintentional injuries among children. Parents could freely communicate with the other members, and the community childcare doctor was not required to answer any questions unless she wished to do so.

This study was approved (IRB No. 2018-01-0663) by the Medical Research Ethics Committee, School of Public Health, Fudan University and registered at Chinese Clinical Trial Registry on 17 January, 2019. All participants provided informed consent prior to participation.

### 2.2. Sample size

We calculated sample size using a formula for comparing two incidences at a 1:1 ratio (representing the intervention group and control group, respectively); unintentional injury rate was used as the indicator. The formula was:


$$\begin{array}{l} n_{1}=n_{2}=\frac{{\left[Z_{\alpha }\sqrt{2P\left(1-P\right)}+Z_{\beta }\sqrt{P_{1}\left(1-P_{1}\right)+P_{2}\left(1-P_{2}\right)}\right]}^{2}}{{\left(P_{1}-P_{2}\right)}^{2}},\\ P=\frac{P_{1}+P_{2}}{2}\;(46). \end{array}$$

In a previous non-controlled intervention study conducted in China, on-site research focusing on children aged 1–6 years found unintentional injury rates of 23% and 4% before and after the intervention, respectively (47). We used the following values for the sample-size calculation: α = 0.05, β = 0.20, P1 = 0.10, P2 = 0.23, which indicated that each group should comprise at least 125 parents. Assuming a dropout rate of 10–50% (based on typical rates for online interventions) (48, 49), this meant that 138–188 parents were required for each group. All sample-size calculations were completed using PASS 2021.

### 2.3. Participants

According to the Children Health Management System, the central area of Jiading District contains 38 resident committees, and ~3,500 children aged 0–3 years who are registered with the child-care-management system of the local community health service centers and who receive regular physical examinations. There are two towns in the area, and each town has one health community center (Jiading Town Health Community Center and Juyuan Town Health Community Center, respectively). From January 1 to February 28, 2019, parents who brought their children to the community health service centers to receive physical examinations were asked to participate in this study, and those that agreed were allocated to one of four WeChat groups. Stratified sampling by child age was used for recruitment. In each town, the parents who agreed to participate were randomly allocated to an intervention group or a control group using a random number table. Thus, the following four WeChat groups were consequently created: Jiading invention group and Jiading control group, and Juyuan invention group and Juyuan control group, respectively. The present study analyzed data for the Jiading intervention group and the Jiading control group. The socio-demographic characteristics comparison and the time-series plot of chatting records were shown in Supplementary Tables S1–S7 and Supplementary Figures S1–S3.

The inclusion criteria for parents were as follows: (1) having a child aged 0–3 years; (2) being the main child caregiver (also eligible if other parent was the main child caregiver) who cares child in daily life and spends much time on caring; (3) being a frequent user of WeChat who uses it in daily life and spends much time on it; (4) having the ability of reading and writing; (5) willing to voluntarily provide informed consent; (6) not planning to move residence from March 2019 to December 2020.

The exclusion criteria for parents were as follows: (1) providing missing data for children about importance indicators and cannot be filled; (2) not cooperating with community childcare doctors in regard to completing their child's treatment; (3) the presence of a disability among the child or parents; (4) participating (the parents and/or their children) in another study.

### 2.4. Patient and public involvement

No patients or members of the public were involved in the design, conduct, reporting, or dissemination of this research.

### 2.5. Processing

Before the establishment of the WeChat groups, the research team created 30 educational articles concerning five potential unintentional injuries and parents' beliefs and skills (of varying severity and susceptibility) among children (33). Among of them, three articles about falls, five articles about burns, five articles about drowning, three articles about poisoning, five articles about asphyxia, and nine articles about parents' beliefs and skills (see Supplementary Table S11). The injuries were chosen through expert consultation based on the Haddon matrix and parents' existing skills and knowledge. An official WeChat account named *Child Safety and Health* was established, and all articles were delivered through this platform. To prevent intergroup pollution, the official WeChat account was set as a private platform and was accessible only to the parents from the intervention groups. The control group members could not search for the group or follow it. The four WeChat groups were established on March 1, 2019, and all members were added individually by the assistant to the community childcare doctor.

Both intervention groups comprised the participating parents, a community childcare doctor, and the doctor's assistant. All parents were asked to follow the official WeChat account and, over the first 3 months, the assistant regularly uploaded articles and sent relevant messages and URLs to the groups. Additionally, all parents could ask the community childcare doctor questions about childcare, and they could also exchange perspectives with other parents and attempt to answer other parents' questions.

Both control groups had the same member composition as the intervention groups, but the parents were not asked to follow the official WeChat account and the community childcare doctor and assistant did not send them relevant messages and URLs. However, due to ethics requirements, the community childcare doctor did answer any emergency questions from the parents.

Finally, all communication records were exported to Excel 2019 through Python, and all group members were given unique numbers; the assistant was numbered “8888,” and the community childcare doctor was numbered “9999.” All records were screened and paired based on criteria of interaction that were used to determine each group member's engagement with the research contents (Supplementary Table S12).

All communication records were exported as a text file, and input in an Excel file to form interactive pairs based on Supplementary Table S12. They were imported into a text file to generate an interactive matrix in the Ucinet 6 data language. As all interactions in each group were shaped by all group members in each group, the social networks for this study represented one-mode directed networks, which meant that the nodes for each row and column were the same (50). All of the indicators listed below were calculated using Ucinet 6.

### 2.6. Statistical analysis

All sociodemographic statistics were analyzed using Stata 17.0. Continuous variables were described using means (95% confidence intervals) and tested using a two independent samples t-test (for continuous variables which are normal distribution, such as parents' age). Discrete variables were described using n (%) and tested using a two independent samples chi-square test (for categories variables without ranks) and Wilcoxon test (for categories variables with ranks).

SNA, which was completed by Ucinet 6, includes both ego network analysis and whole network analysis (50, 51). Hypothesis H_01_ could be tested through ego network analysis; Hypothesis H_02_ could be tested using the density, distance, centrality, and cohesion subgroups; and, finally, hypothesis H_03_ could be tested using structural hole. The interpretations of them were listed in Supplementary material.

#### 2.6.1. Ego network

##### 2.6.1.1. Coreness

Coreness reflects the degree of activity of each group member, and can be used to divide group members into five ranks: core, active, little activity, silent, and alienated (for details, see Supplementary Table S13) (52). Coreness was calculated using the following path: “Network → Core/Periphery → Continuous.” For this calculation, the inputted data should follow an adjacency matrix that contains only 0 and 1.

##### 2.6.1.2. Basic ego measures

Basic measures for ego comprise 14 indicators. Among these, the size of the ego network, the number of directed ties, the network's density, its two-step reach, and the reach efficiency are important. For Parent A, the size of the ego network represents the number of other parents directly linked with Parent A, including Parent A himself; the number of directed ties represents all ties (Supplementary Table S12) in Parent A's ego network; density represents number of actual ties divided by ties in theory; two-step reach represents the percentage of actors in the network who are within two steps; and reach efficiency represents two-step reach divided by the network size, which provides standardized data. The above indicators can be calculated using the following path: “Network → Ego networks → Basic ego measures.” The input data should be in the form of an adjacency matrix.

#### 2.6.2. Whole network

##### 2.6.2.1. Density

If two parents have mutual communications, they have a reciprocal relationship (53); if all members have reciprocal relationships, the social network is named a “complete network” (50). Density is an indicator that reflects whether group members have a close association with other members (54). It can be calculated by dividing the number of relationships in fact by the number of relationships in theory. For a directed network, we can suppose that each members' number is “*n*” and the number of relationships in fact is “m”; thus, the number of relationships in theory is “*n*(*n*-1)” and the formula for the density is $\frac{m}{n\left(n-1\right)}$. Density can be calculated using the following path: “Network → Cohesion → Density → Overall Density.” The input data should be in the form of an origin matrix. Greater values here mean more interconnections.

##### 2.6.2.2. Distance

Distance indicates the length of the shortest path between any two nodes in the social network. Three indicators are included: average distance, distance-based cohesion (compactness), and distance-weighted fragmentation (50). The present study focused on distance-based cohesion (compactness), which represents the average of all multiplicative inverses determined based on each distance in the distance matrix, and demonstrates the degree of closeness between all social-network members. Larger values indicate more closeness and more cohesion. Density can be calculated using the following path: “Network → Cohesion → Geodesic distance.” The input data should be in the form of an adjacency matrix.

##### 2.6.2.3. Centrality

Centrality was used to demonstrate, for each WeChat group member, whether they held a central position in the group and the level of power they had. Centrality is based on three kinds of indicators: point centrality, betweenness centrality, and closeness centrality. In the Ucinet 6, the input data should be in the form of an adjacency matrix.

###### 2.6.2.3.1. Point centrality

Point centrality means the number of other parents who directly link with Parent A; a bigger value indicates a more central position (55). Additionally, for directed social networks the point centrality of each parent can be divided into in-degree centrality, which represents the number of connections received, and out-degree centrality, which represents the number of connections delivered. Point centrality can be calculated using the following path: “Network → Centrality → Degree”; data should not be treated as symmetric.

###### 2.6.2.3.2. Betweenness centrality

Betweenness centrality demonstrates the degree that Parent A can control the communication between other parents, or between other parents and the community childcare doctor. In a social network, this indicator represents the geodesic distance between Parent A and other pairs of parents. Bigger values indicate higher degrees of control of communication between other parents or between other parents and the community childcare doctor (55). Betweenness centrality can be calculated using the following path: “Network → Centrality → Freeman betweenness → Node betweenness.”

###### 2.6.2.3.3. Closeness centrality

Closeness centrality represents the degree to which Parent A is not controlled by other members. This indicator is calculated by determining the sum of the geodesic distances between Parent A and the other parents and between Parent A and the community childcare doctor. Higher values indicate further distance from the core of the social network (55). Closeness centrality can be calculated using the following path: “Network → Centrality → Closeness.”

##### 2.6.2.4. Cohesion subgroups

Cohesion subgroups reflect the substructure of each WeChat group. To consider the social network's characteristics, for the present study we based on reciprocal relationships (56). This perspective included two aspects: components and cliques.

###### 2.6.2.4.1. Components

If any two group members can be connected by a certain pathway across a set of points, this pathway is called a “component.” There are two kinds of components: strong, which relates to a directed graph, and weak, which relates to an undirected graph (57). Components can be measured using the following path: “Network → Regions → Components → Simple graphs,” with both weak and strong component types being considered. The input data should be in the form of an adjacency matrix.

###### 2.6.2.4.2. Cliques

Cliques indicate that the relationships between all members in a subgroup are reciprocal, and that no new member can be added to the subgroup (56). A clique represents the most basic cohesion subgroup, and can be measured using the following path: “Network → Subgroups → Cliques. The input data should be in the form of a symmetric adjacency matrix. To reveal possibly hidden cliques, decrease the number of cliques, and/or determine the main members, outsiders, and leaders in a clique, a co-membership matrix is used, which outputs data in the form of a “clique-by-clique actor co-membership matrix.”

##### 2.6.2.5. Structural hole

Structural holes were first suggested by Burt, and were used in this study to identify non-redundant relationships between two parents (58). Supplementary Figure 4 provides details regarding the redundant and non-redundant relationships in the network. Supplementary Figure S4a shows a structural hole in which ego has an association not only with Parent A, but also with Parent B, while Parent A and Parent B have no relationship; this represents a hole because ego must deliver information to parent A and parent B, respectively. In Supplementary Figure S4b, a structural hole is not present because Parent A has an association with Parent B, and ego only needs to deliver information to Parent A, who can then provide the information to Parent B; thus, the relationship between ego and Parent B is redundant.

There are four important indicators for structural holes: (1) the effective size of the network: this indicator represents the number of non-redundant factors in Parent A's network; (2) efficiency: for Parent A, this indicator represents the ratio of effective size to actual size; (3) constraint: this demonstrates the impact of the presence of structural holes in an ego network; and (4) hierarchy: this demonstrates the extent to which constraint focuses on each parent and the community childcare doctor. Among these, the effective size of the network and constraint are very important. Structural holes can be calculated using the following path: “Network → Ego Networks → Structural holes,” with “whole network model—normal method” selected as the method. The input data should be in the form of a symmetric adjacency matrix.

## 3. Results

### 3.1. Characteristics of the study population

Seventy-four parents were enrolled in the Jiading intervention group, and 64 parents were enrolled in the Jiading control group. There were no statistical differences between the intervention group and the control group in terms of sociodemographic characteristics (Supplementary Table S2). Additionally, there were not statistical difference for participants who withdrawn and completed this study in Jiading and Juyuan (Supplementary Tables S8–S10).

### 3.2. Characteristics of the WeChat groups

**Figure 2:**
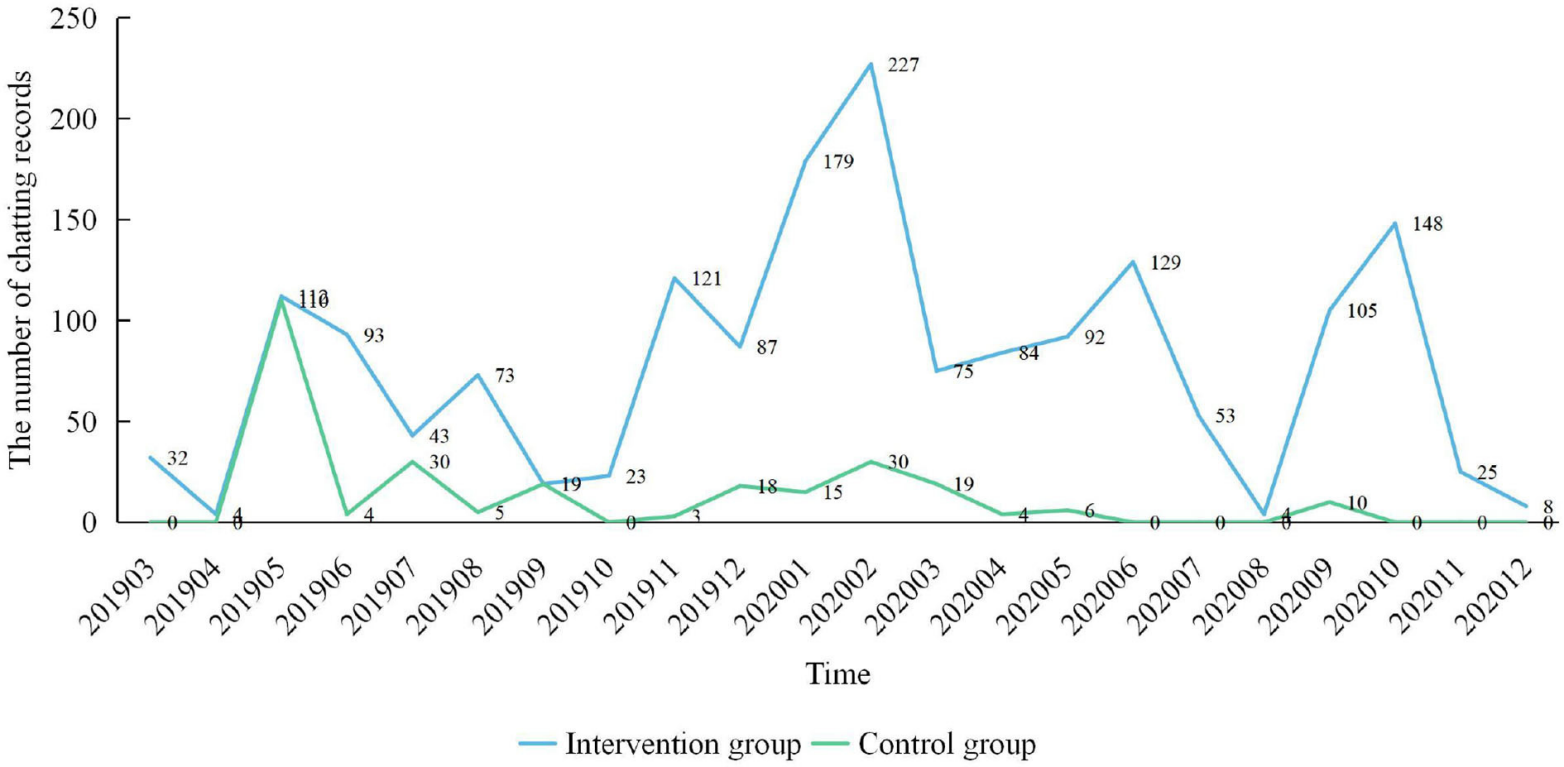
The time-series plot of intervention group and control group chatting records.

Including the community childcare doctor and the assistant, 66 and 76 members were included in the control and intervention groups, respectively. In the control group, 11 members (31.9%) communicated during the intervention and follow-up and 273 communication records were obtained. In the intervention group, 49 members (64.5%) communicated and 1,736 communication records were obtained. A time-series plot (Figure 2) showed that the intervention group was more active than the control group, which indicates that a health-education program based on a WeChat group may enhance parents' activity in regard to (1) obtaining knowledge about children aged 0–3 years, (2) exchanging their thoughts with other parents and community childcare doctors, and (3) taking the initiative in regard to asking community childcare doctors questions.

### 3.3. Ego network analysis

In the control group, 17 members (25.8%) were identified as core members, three members (4.5%) were active, and 46 members (69.7%) were alienated. In the intervention group, 15 members (22.7%) were core members, 33 (50.0%) were active, one member (1.5%) was showed little activity, and 27 members (40.9%) were alienated.

The community childcare doctor showed that the ego network (the size value was 17 and 44 in the control and intervention groups, respectively), had the largest number of ties (the ties value was 14 and 128 in the control and intervention groups, respectively), but the smallest density (the density value was 5.15% and 6.77% in the control and intervention groups, respectively), which demonstrated that the community childcare doctor was positioned in a core situation, mainly received (rather than sent) parents' messages, and was responsible for answering parents' questions. In addition, the two-step reach of the community childcare doctor was 29.23% and 65.33% in the control and intervention group, respectively, which was relatively high in all ego networks, and which demonstrates that the community childcare doctor had an ability to deliver information regarding the safety of children aged 0–3.

For the other parents, in the control group four parents (parent 1104, parent 1128, parent 1145 and parent 1200) had sizes of 6, 5, 4, and 3, respectively; the values for their ties were 8, 7, 4, and 2, respectively, their density values were 26.67%, 35.00%, 33.33%, and 33.33%, respectively; and their two-step reach values were 32.31%, 29.23%, 32.31%, and 26.15%, respectively. This demonstrated that they played an active role, not only asking questions of the community childcare doctor, but also answering other parents' questions or providing suggestions. Additionally, they, as core WeChat group members, participated in most interactions.

In the intervention group, 14 parents (parent 1119, parent 1074, parent 1196, parent 1004, parent 1146, parent 1222, parent 1058, parent 1241, parent 1056, parent 1080, parent 1109, parent 1189, parent 1218 and parent 1174) showed sizes of 19, 17, 15, 13, 12, 11, 11, 10, 10, 9, 8, 8, 7, and 7, respectively; the values for their ties were 95, 95, 82, 56, 45, 41, 61, 46, 49, 48, 35, 12, 28, and 19, respectively; their density values were 27.78%, 34.93%, 39.05%, 35.90%, 34.09%, 37.27%, 55.45%, 51.11%, 54.44%, 66.67%, 62.50%, 21.43%, 66.67%, and 45.24%, respectively, and their two-step reach values were 64.00%, 64.00%, 65.33%, 64.00%, 64.00%, 64.00%, 62.67%, 64.00%, 61.33%, 61.33%, 61.33%, 61.33%, 62.67%, and 37.33%, respectively. This suggests that these parents played an important role in helping the community childcare doctor answer some of the other parents' questions.

Compared to the control group, the intervention group was more active and featured more parents who may have played a role in answering questions, which shaped its post-intervention ego network (Supplementary Tables S13, S14).

### 3.4. Whole network analysis

**Figure 3:**
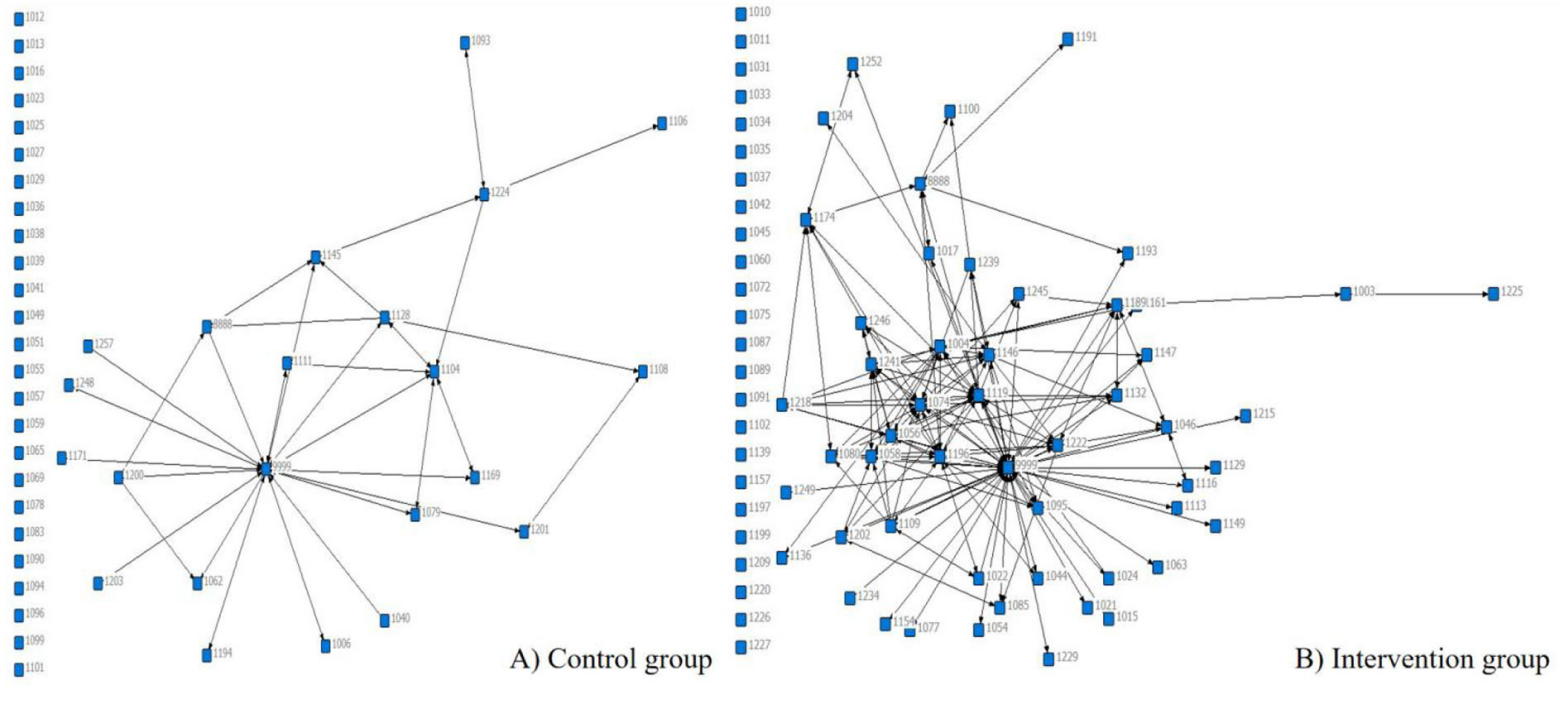
The social network of intervention group and control group.

Figure 3 shows social network graphs for the control and intervention groups. Besides the community childcare doctor, who was numbered “9999,” all parents who were mentioned above as being at the core of their social networks and who served as leaders were responsible for sharing the community childcare doctor's workload in terms of answering questions from parents in the group. Although both the control and intervention group featured parents who served as core members, the control group network was less close than the intervention group network. All of the following results are based on the social network graphs.

#### 3.4.1. Density and distance

For the control group, the density was 0.0590 and the distance-based cohesion (compactness) was 4.4%. For the intervention group, the density was 0.2912 and the distance-based cohesion (compactness) was 21.6%. Compared to the control group, the intervention group had ~ five-times the density and compactness, which demonstrated that the communication in the intervention group was closer and that more interactions occurred between parents. This indicated that the online-social-network-based parental health education intervention has a positive impact on enhancing parents' knowledge, beliefs, and skills about child unintentional injuries (see Supplementary material).

#### 3.4.2. Centrality

##### 3.4.2.1. Point centrality

In the control group, the community childcare doctor had the largest in-degree centrality, 16, and out-degree centrality, 13, which was ~ three times that of the other parents. In addition, all group members had approximately the same in-degree and out-degree centrality values, which demonstrated that the parents mainly communicated only with the doctor (Supplementary Table S16).

In the intervention group, the overall situation was similar to the control group. However, parent 1196, parent 1004, parent 1218, and parent 1109 showed greater out-degree centrality than in-degree centrality, which demonstrated that they were willing to help other parents in regard to answering their questions. Meanwhile, parent 1056, parent 1058, and parent 1080, showed greater in-degree centrality than out-degree centrality, which demonstrated that they frequently received help from other parents (Supplementary Table S17).

##### 3.4.2.2. Betweenness centrality

In the control group, the community childcare doctor had the largest betweenness centrality, 256.5; meanwhile, parent 1145 and parent 1224 had the largest betweenness centrality when compared with other parents (64.7 and 53.5, respectively), which demonstrated that they had acquired considerable information about children aged 0–3, and could play an important role in regard to facilitating communication among group members (Supplementary Table S16).

In the intervention group, parent 1003 showed the largest betweenness centrality (1,706.5), while parent 1004, parent 1010, parent 1011, parent 1015, and parent 1017 also showed larger betweenness centralities than the other parents (157.0, 133.9, 122.6, 115.0, and 112.5, respectively). The community childcare doctor's betweenness centrality was 0, which demonstrated that, after receiving the intervention, some parents could resolve certain problems through mutual communication (Supplementary Table S17).

##### 3.4.2.3. Closeness centrality

In the control and intervention groups, all group members' in-closeness centrality and out-closeness centrality were the same and small, at almost 2 and 3, respectively, which demonstrated that the social network was loose and had multiple centers (Supplementary Tables S16, S17).

#### 3.4.3. Cohesion subgroups

##### 3.4.3.1. Components

Regarding weak components among the group members who participated in the social communication network, the control and intervention groups featured a component that included 22 nodes and 52 nodes, respectively, and the proportion was 33.3% and 68.4%, respectively (Tables 1, 2).

**Table 1:** The component of control group social network nodes except isolation nodes.

**Type**	**Components**	**Nodes**	**Proportion**
Weak	1	1006, 1040, 1062, 1079, 1093, 1104, 1106, 1108, 1111, 1128, 1145, 1169, 1171, 1194, 1200, 1201, 1203, 1224, 1248, 1257, 8888, 9999	0.333
Strong	1	1006, 1079, 1093, 1104, 1108, 1111, 1128, 1145, 1169, 1171, 1194, 1200, 1201, 1224, 1248, 8888, 9999	0.258
	2	1040	0.015
	3	1062	0.015
	4	1106	0.015
	5	1203	0.015
	6	1257	0.015

^*^For weak and strong components, other isolated nodes' proportion was 0.015.

**Table 2:** The component of intervention group social network nodes except isolation nodes.

**Type**	**Components**	**Nodes**	**Proportion**
Weak	1	1003, 1004, 1015, 1017, 1021, 1022, 1024, 1044, 1046, 1054, 1056, 1058, 1063, 1074, 1077, 1080, 1085, 1095, 1100, 1109, 1113, 1116, 1119, 1129, 1132, 1136, 1146, 1147, 1149, 1154, 1161, 1174, 1189, 1191, 1193, 1196, 1202, 1204, 1215, 1218, 1222, 1225, 1229, 1234, 1239, 1241, 1245, 1246, 1249, 1252, 8888, 9999	0.684
Strong	1	1003, 1004, 1015, 1017, 1022, 1024, 1044, 1046, 1054, 1056, 1058, 1063, 1074, 1077, 1080, 1085, 1095, 1109, 1113, 1116, 1119, 1129, 1132, 1136, 1146, 1147, 1149, 1154, 1161, 1174, 1189, 1191, 1193, 1196, 1202, 1204, 1215, 1218, 1222, 1229, 1239, 1241, 1245, 1246, 1252, 8888, 9999	0.618
	2	1021	0.013
	3	1100	0.013
	4	1225	0.013
	5	1234	0.013
	6	1249	0.013

^*^For weak and strong components, other isolated nodes' proportion was 0.013.

There were six strong components in the control group; among these, one component included 17 nodes and the proportion was 25.8%; the other components were parent 1040, parent 1062, parent 1106, parent 1203, and parent 1257, respectively, and the proportion was 1.5%. For the intervention group, there were six strong components; among these, one component included 47 nodes and the proportion was 61.8%; the other components were parent 1021, parent 1100, parent 1225, parent 1234, and parent 1249, respectively, and the overall proportion was 1.3%. This demonstrated that they showed inactive communication with others (Tables 1, 2).

##### 3.4.3.2. Cliques

For the control group, four cliques were found: “1079, 1104, and 9999,” “1104, 1111, and 9999,” “1104, 1128, and 9999,” and “1104, 1169, 9999,” respectively. The other parents were not included in any cliques. Based on a “clique-by-clique actor co-membership matrix,” the cliques could combine to form a large clique comprising “1079, 1104, 9999, 1111, 1128, and 1169”; the core of this clique was “1104 and 9999” (Table 3).

**Table 3:** The cliques of control group.

**Cliques number**	**Nodes**
1	1079, 1104, 9999
2	1104, 1111, 9999
3	1104, 1128, 9999
4	1104, 1169, 9999

For the intervention group, 28 cliques were found, comprising a total of 27 group members' the other parents were not included in any cliques. Based on the “clique-by-clique actor co-membership matrix,” the cliques could be combined to form a large clique comprising “9999, 1074, 1119, 1196, 1004, 1189, 1241, 1056, 1109, 1222, 1080, 1132, 1146, 1174, 1202, 1245, 8888, 1022, 1044, 1058, 1085, 1095, 1147, 1161, 1218, 1239, and 1246.” Here, the main members were “9999, 1074, 1119, and 1004” (Table 4).

**Table 4:** The cliques of intervention group.

**Cliques number**	**Nodes**	**Cliques number**	**Nodes**
1	1074, 1080, 1119, 1196, 9999	15	1085, 1202, 9999
2	1004, 1074, 1119, 1196, 9999	16	1109, 1196, 9999
3	1074, 1119, 1196, 1241, 9999	17	1109, 1222, 9999
4	1058, 1074, 1119, 1241, 9999	18	1004, 1147, 9999
5	1074, 1119, 1222, 9999	19	1004, 1161, 9999
6	1074, 1132, 9999	20	1132, 1189, 9999
7	1004, 1074, 1146, 9999	21	1004, 1189, 9999
8	1074, 1202, 9999	22	1189, 1222, 9999
9	1004, 1074, 1218, 9999	23	1189, 1245, 9999
10	1074, 1245, 9999	24	1146, 1239, 9999
11	1044, 1196, 9999	25	1022, 1109, 1196
12	1056, 1119, 1196, 1241, 9999	26	1074, 1080, 1119, 1174
13	1056, 1119, 1241, 1246, 9999	27	1119, 1174, 8888
14	1056, 1095, 9999	28	1119, 1196, 8888

Although, according to the “clique-by-clique actor co-membership matrix,” both the control and intervention group formed large cliques, when compared to the control group the intervention group featured more parents who served as core members and who participated in many cliques. For the control group, the community childcare doctor controlled virtually all communications and answered virtually all of the parents' questions. For the intervention group, more parents participated in communications in the post-intervention period.

From the perspective of their education, high education level (college or above) occurred in some cliques usually, which demonstrated that high education level parents may serve as child-unintentional-injuries-related information transporter in different subgroups. For community childcare doctor, they should arouse high education level parents activity and passion of studying child-unintentional-injuries-related information and teaching other parents according to their understand and exercise.

#### 3.4.4. Structural hole

Tables 5, 6 show, for the control and intervention groups, effective size and constraint, respectively.

**Table 5:** The structural hoes of control group.

**Number**	**ID**	**EffSize**	**Constraint**	**Number**	**ID**	**EffSize**	**Constraint**	**Number**	**ID**	**EffSize**	**Constraint**
1	1006	1.000	1.000	23	1079	1.000	0.635	45	1160	0.000	0.000
2	1012	0.000	0.000	24	1083	0.000	0.000	46	1167	0.000	0.000
3	1013	0.000	0.000	25	1090	0.000	0.000	47	1169	1.000	0.635
4	1016	0.000	0.000	26	1093	1.000	1.000	48	1170	0.000	0.000
5	1023	0.000	0.000	27	1094	0.000	0.000	49	1171	1.000	1.000
6	1025	0.000	0.000	28	1096	0.000	0.000	50	1173	0.000	0.000
7	1027	0.000	0.000	29	1099	0.000	0.000	51	1180	0.000	0.000
8	1029	0.000	0.000	30	1101	0.000	0.000	52	1190	0.000	0.000
9	1036	0.000	0.000	31	1103	0.000	0.000	53	1194	1.000	1.000
10	1038	0.000	0.000	32	1104	4.545	0.409	54	1198	0.000	0.000
11	1039	0.000	0.000	33	1105	0.000	0.000	55	1200	2.000	0.598
12	1040	1.000	1.000	34	1106	1.000	1.000	56	1201	2.000	0.500
13	1041	0.000	0.000	35	1107	0.000	0.000	57	1203	1.000	1.000
14	1049	0.000	0.000	36	1108	2.000	0.500	58	1212	0.000	0.000
15	1051	0.000	0.000	37	1111	1.000	0.635	59	1223	0.000	0.000
16	1055	0.000	0.000	38	1123	0.000	0.000	60	1224	4.000	0.278
17	1057	0.000	0.000	39	1128	3.688	0.326	61	1230	0.000	0.000
18	1059	0.000	0.000	40	1130	0.000	0.000	62	1248	1.000	1.000
19	1062	1.000	0.848	41	1140	0.000	0.000	63	1250	0.000	0.000
20	1065	0.000	0.000	42	1143	0.000	0.000	64	1257	1.000	1.000
21	1069	0.000	0.000	43	1145	3.071	0.371	65	8888	2.300	0.527
22	1078	0.000	0.000	44	1151	0.000	0.000	66	9999	16.086	0.119

**Table 6:** The structural holes of intervention group.

**Number**	**ID**	**EffSize**	**Constraint**	**Number**	**ID**	**EffSize**	**Constraint**	**Number**	**ID**	**EffSize**	**Constraint**
1	1003	2.000	0.556	27	1077	1.000	1.000	53	1193	2.000	0.556
2	1004	8.545	0.204	28	1080	3.393	0.255	54	1196	9.442	0.189
3	1010	0.000	0.000	29	1085	1.600	0.487	55	1197	0.000	0.000
4	1011	0.000	0.000	30	1087	0.000	0.000	56	1199	0.000	0.000
5	1015	1.000	1.000	31	1089	0.000	0.000	57	1202	2.250	0.348
6	1017	2.000	0.500	32	1091	0.000	0.000	58	1204	1.000	1.000
7	1021	1.000	1.000	33	1095	2.286	0.321	59	1209	0.000	0.000
8	1022	1.000	0.501	34	1100	2.000	0.500	60	1215	1.000	1.000
9	1024	1.000	0.596	35	1102	0.000	0.000	61	1218	2.700	0.273
10	1031	0.000	0.000	36	1109	3.667	0.286	62	1220	0.000	0.000
11	1033	0.000	0.000	37	1113	1.000	1.000	63	1222	6.938	0.204
12	1034	0.000	0.000	38	1116	1.500	0.583	64	1225	1.000	1.000
13	1035	0.000	0.000	39	1119	13.387	0.179	65	1226	0.000	0.000
14	1037	0.000	0.000	40	1129	1.000	1.000	66	1227	0.000	0.000
15	1042	0.000	0.000	41	1132	2.438	0.292	67	1229	1.000	1.000
16	1044	1.000	0.552	42	1136	1.000	0.608	68	1234	1.000	1.000
17	1045	0.000	0.000	43	1139	0.000	0.000	69	1239	2.857	0.288
18	1046	3.000	0.368	44	1146	8.000	0.186	70	1241	5.000	0.238
19	1054	1.000	1.000	45	1147	1.600	0.425	71	1245	2.000	0.329
20	1056	5.000	0.257	46	1149	1.000	1.000	72	1246	1.000	0.390
21	1058	4.900	0.243	47	1154	1.000	1.000	73	1249	1.000	1.000
22	1060	0.000	0.000	48	1157	0.000	0.000	74	1252	2.000	0.500
23	1063	1.000	1.000	49	1161	1.000	0.560	75	8888	6.167	0.183
24	1072	0.000	0.000	50	1174	4.292	0.244	76	9999	40.976	0.059
25	1074	11.274	0.180	51	1189	6.467	0.191	–	–	–	–
26	1075	0.000	0.000	52	1191	1.000	1.000	–	–	–	–

##### 3.4.4.1. Effective size

For the control group, 7.5% showed an effective size exceeding 2.000, which indicated that they actively participated in interaction and had strong control over and influence on other parents. The community childcare doctor had the largest effective size, at 16.086, and parent 1104, parent 1224, parent 1128, and parent 1145 showed the largest effective sizes among the parents, at 4.545, 4.000, 3.688, and 3.071, respectively. Seventeen (25.8%) parents had effective sizes between 2.000 and 1.000, which demonstrated that they seldom participated in group interaction. Forty-four (66.7%) parents had an effective size of 0.000, meaning they merely viewed the communications without participating.

For the intervention group, 23.7% of the members had effective sizes larger than 2.000, which indicated that they actively participated in the interactions and had strong control over and influence on other parents. The community childcare doctor had the largest effective size, at 40.976, and among the parents, parent 1119, parent 1074, parent 1196, parent 1004, parent 1146, and parent 1222 had the largest effective sizes, at 13.387, 11.274, 9.442, 8.545, 8.000, 6.938, and 6.467, respectively. Meanwhile, some parents had effective sizes of 3.000–5.000. Thirty-four (44.7%) parents had effective sizes between 2.000 and 1.000, demonstrating that they seldom participated in the group interaction. Twenty-four (31.6%) parents had an effective size of 0.000, meaning they only viewed the communications and did not participate in them.

Comparing the control group to the intervention group showed that, besides the community childcare doctor, more parents from the intervention group improved their knowledge about children aged 0–3 years, and the intervention-group parents' participation activity increased after receiving the health education.

##### 3.4.4.2. Constraint

For group members who had an effective size of ≥1.000, nine (40.9%) from the control group and 16 (30.8%) from the intervention group showed constraint values of 1.000, which demonstrated that they were strictly constrained by the associated group members, showed the largest constraint or dependence, and that their ability to cross-jump structural holes was very weak. For the other group members, the constraint values were < 1.000, which demonstrated that they were strictly constrained by the associated group members, had smaller constraint or dependence, and very strong ability to cross-jump structural holes.

Compared to the control group, the intervention group showed a closer association.

Considering aforementioned indicators comprehensively, in-degree centrality was associated with out-degree centrality in intervention group and control group (rs = 0.939, *P* < 0.001; rs = 0.890, *P* < 0.001), in-closeness centrality was associated with out-closeness centrality in intervention group and control group (rs = 0.745, *P* < 0.001; rs = 0.716, *P* < 0.001). It demonstrated that in social network, if parents received much more information from other parents, they will also deliver much more information to other parents so that the communications between parents get strengthened. For aforementioned parents (4 parents in control group and 14 parents in intervention group) who play active role in social network to help community childcare doctor to deliver child-unintentional-injuries-related information and teach other parents, 86.1% parents' education level was college or above, 11.1% parents' education level was senior high school, 2.8% parents' education level was middle school. Percent 25 parents were other and unemployed, they have much more time to care their child.

Above all, high education level parents may serve as community childcare doctors' assistant to provide service or response to other parents. In community parental health education intervention, community health workers can inspire parents' motivation to study child-unintentional-injuries-related information and participate in community health management by implementing health education on high education level parents in the community social network. Additionally, from the perspective of childcare, community health workers should focus on parents who have much more time to accompany with their child and can calculate a certain experience about childcare.

## 4. Discussion

The present study aimed to explore the mechanisms underlying the effect of online-social-media-based parental health-education interventions for improving children's health status and parents' associated knowledge and skills. To perform this exploration, SNA was applied. Comparing the outcomes for the intervention group with those for the control group provided the following main results:

Regarding hypothesis H_01_, some parents (four parents in the control group and 14 parents in the intervention group) who acquired better knowledge about childcare voluntarily sought to answer other parents' questions, which was effective for relieving the community childcare doctor's workload. Most of these parents were mothers who were married, usually worked in Shanghai, and had undergraduate or higher education level; this suggests that they were better able to locate and acquire knowledge about childcare and to care for children. Comparing the number of core parents in the two groups showed that the intervention group had approximately four times the number of those in the control group, which suggested that the intervention and the communication with the community childcare doctor effectively enhanced the knowledge of the parents in the intervention group. Hence, if parents acquire more knowledge, their roles may transfer from information recipient to a combination of information recipient and sender, and they may then more actively participate in interactions.

Regarding hypothesis H_02_, the intervention conducted by the doctor and assistant encouraged more parents to participate in interactions between group members. The interactions of the intervention group members were closer than those of the control group members, and the intervention group's centrality also improved significantly when compared to the control group. Comment frequencies and comment days also improved significantly. First, because the doctor and assistant were professionals, if parents had questions they could receive answers quickly from the doctor. Second, the design of the intervention tool focused mainly on potential unintentional injuries and aspects that can be easily missed by parents, meaning the intervention could arouse parents' enthusiasm to participate in interactions. Third, in the WeChat groups parents' questions are visible to all group members, meaning if a parent felt that a question concerned a topic related to their own child, they could participate in the interaction. Additionally, most of the parents were ~30 years old, had a high education level, and were likely to have had discussions with their parents regarding childcare, meaning they were likely to be relatively accustomed to intense discussions on childcare topics. The parents were able to reach a consensus on some aspects by showing their agreement with the community childcare doctor, and this may have encouraged the parents to engage in further study beyond the intervention articles. This indicates that the WeChat-group-based intervention could strengthen and tighten communication between parents and community childcare doctors and between parents and other parents.

Regarding hypothesis H_03_, a bridge was created between parents, which enhanced their acquiring of childcare knowledge. As only one community childcare doctor was included in the WeChat group, it was difficult to rely solely on communication with the childcare doctor to receive a timely answer to certain questions. After receiving health education, the parents from the intervention group showed a larger effective size and stronger ability to cross-jump structural holes. Through observing the results for centrality, especially betweenness centrality, it was deemed that, although the social networks had multiple centers, the intervention group contained more parents who served as bridges for conveying information. Additionally, according with the aforementioned hypothesis, if other parents asked similar questions these parents were willing to provide answers voluntarily. Through this, the parents shared the doctor's responsibility. Hence, the WeChat-group-based parental health education served to build a bridge to promote interaction between parents rather than merely between parents and the doctor.

From the perspective of the intervention platform and tools, the present study used WeChat as the intervention platform. WeChat is not only free, but is also used widely in China to communicate with others one-to-one or in groups. In addition, this application can help people search for specific information. WeChat has similar functions to Facebook and Twitter, which are widely used in Western countries such as America; this is notable because these social-network systems have been found to potentially be effective for providing health education and for sharing information with others (31). A previous cluster randomized controlled trial explored an app-based unintentional-injuries-prevention intervention that featured a similar design to the intervention tool used in the present study (e.g., the Haddon matrix) (32). The study found that the health education platform and tools were effective for reducing the incidence of unintentional injuries among children.

From the perspective of SNA, some studies of community-based interventions have found that the intervention information can be diffused through social interactions, such as active and voluntary communication with other group members; this is similar to the observations of the present study (59, 60). Further, a previous investigation of a community-based parenting intervention that featured children's primary caregivers as subjects stated that community mobilization is an effective tool for helping children's primary caregivers study how to prevent and reduce health and social risk factors (60). In the present study, health education was conducted through WeChat groups and the intervention articles delivered by the doctor's assistant, with some general introductions to the main content of the articles being included. This process helped to motivate parents to study and acquire related knowledge, which also improved their skills.

The health education conducted in this study was in an mHealth format, and the strengths of this health-education program and study were as follows:

First, this health-education program could shorten social distance. Social distance, one of the components of psychological distance (61), means the degree to which individuals or groups are excluded by others (62). It can influence not only the degree of trust between people, but also their information receptiveness and mutual emotions (63). The present study concluded that a WeChat-group-based intervention can not only eliminate barriers of time and space, but also afford direct communication with others and with community childcare doctors. In addition, this intervention was conducted during the Coronavirus Disease 2019 Pandemic, a time when many people were isolated at home and may have had difficulties attending health-care facilities; mHealth and telemedicine health education represent possible means of mitigating this issue.

Second, health education could save community childcare doctors time. Through expert interviews, the community childcare doctor stated that the health education allowed more parents to acquire childcare knowledge and improve their knowledge and skills relating to preventing children from acquiring injuries. Concurrently, the intervention also encouraged parents to answer other parents' questions, leaving the community childcare doctor to merely judge whether the parents' answers were correct and sufficiently detailed.

Third, the WeChat-group-based parental-health-education intervention imitated real-world communication, lending it a certain sense of realness.

Fourth, this is the first study to explore the mechanism of an online-social-network-based parental-health education intervention designed to reduce the incidence of unintentional injuries among children, and it was found that such an intervention can enhance parents' knowledge, attitudes, and behaviors in this regard.

In contrast, the study also had some limitations:

First, the study featured social desirability bias. This study was conducted in Shanghai, China, where parents generally have a higher level of education (64); this means that, when compared to people from other areas of China (e.g., northern cities), it may have been easier for these parents to learn the content and answer questions in the WeChat groups.

Second, because the health-education platform was online social media, to access the health-education platform a certain level of regional economic development was necessary.

Third, to assess the mechanisms of health education and their effectiveness, two groups were established, a WeChat group containing parents only and a WeChat group that featured parents and a community childcare doctor. However, the parents-only WeChat group still provided a low level of health education. The reason we did not create a blank control is that it is difficult to confirm parents' issues and questions when examining off-line health education.

The interaction of the intervention group was closer than that of the control group, which may indicate that the effectiveness of the health education originates from communication between parents and/or from communication between parents and the community childcare doctor. In short, although SNA is seldom used to analyze the mechanism of unintentional-injury prevention, the present study obtains the same conclusions as similar previous studies (31): that online social community-based health education *via* social media is an effective way to generally improve health status. The difference between this previous study and the study was study design. The study validated the effectiveness of online social media-based intervention which established two online social communities by randomized controlled trial, which provided a strong evidence to indicate that the implementation of health education through online social communities can be generalized.

## 5. Conclusions

The interaction of the intervention group was closer than that of the control group, which could indicate that the effectiveness of the health education originates from communication between parents and/or between parents and the community childcare doctor. In short, although SNA has seldom been used to analyze the mechanism of unintentional-injury prevention, compared with similar studies the present study obtains the same conclusions: that online-social-community-based health education *via* social media is an effective way to improve general health status. This also indicates that implementing health education through online social communities can be generalized. Parents who received the intervention formed a habit of consulting the community childcare doctor if they had questions regarding childcare. This phenomenon indicates that establishing an online social community that gathers parents in a group and inviting community childcare doctors to participate in the group to provide timely help can foster community interaction that can enhance parents' childcare-related activity. Further, the childcare doctor can also observe inter-parent communication and play a core role in terms of providing education, improving degree of participation, and arousing enthusiasm for group interaction.

## Data availability statement

The raw data supporting the conclusions of this article will be made available by the authors, without undue reservation.

## Ethics statement

The studies involving human participants were reviewed and approved by the Medical Research Ethics Committee, School of Public Health, Fudan University. The patients/participants provided their written informed consent to participate in this study.

## Author contributions

YF contributed to data acquisition, analysis, interpretation, drafting, and revision of the manuscript. XL contributed to data acquisition, interpretation, conception, design, and revision of the manuscript. XM contributed to data acquisition, design, and revision of the manuscript. JG and JX contributed to providing some suggestions on study design, questionnaire, and intervention. RJ contributed to study design and suggested the intervention. ZZ and KC contributed to the analysis of words frequencies. JL contributed to the design and revision of the manuscript. All authors contributed to the article and approved the submitted version.
