# Supplementary material for: Using online social networks to provide a parental health-education intervention for preventing unintentional injuries among children aged 0–3 years: A randomized controlled trial and social network analysis in Shanghai, China
Source: Front Public Health. 2023 Jan 11;10:1049851. doi: 10.3389/fpubh.2022.1049851 (PMC9875045; doi:10.3389/fpubh.2022.1049851)
Supplement: Supplementary file 1 [file Data_Sheet_1.docx]

Supplementary Material

# The socio-demographic characteristics comparison of Jiading and Juyuan

Table S1 The socio-demographic characters comparing Jiading with Juyuan

| Variable | Jiading  n=138 | Juyuan  n=148 | *P* value |
| --- | --- | --- | --- |
| Children age, n (%) ^a^ |  |  | 0.092 |
| 0 | 44 (31.9) | 57 (38.5) |  |
| 1 | 38 (27.5) | 46 (31.1) |  |
| 2 | 29 (21.0) | 24 (16.2) |  |
| 3 | 27 (19.6) | 21 (14.2) |  |
| Father age, y, mean (SD) ^b^ | 33.8 (5.1) | 33.3 (4.3) | 0.356 |
| Father education, n (%) ^a^ |  |  | 0.800 |
| Middle school | 2 (1.5) | 6 (4.1) |  |
| Senior High school | 12 (8.7) | 10 (6.8) |  |
| College or above | 124 (89.9) | 132 (89.2) |  |
| Father job, n (%) ^c^ |  |  | 0.747 |
| Employees of state-owned enterprises and public institutions | 48 (34.8) | 49 (33.1) |  |
| Employees of foreign-funded, private and enterprises | 66 (47.8) | 68 (46.0) |  |
| Other (farmers, soldiers, freelancers) | 24 (17.4) | 31 (21.0) |  |
| Unemployed | 0 (0.0) | 0 (0.0) |  |
| Mother age, y, mean (SD) ^b^ | 31.8 (4.2) | 31.7 (4.1) | 0.925 |
| Mother education, n (%) ^a^ |  |  | 0.762 |
| Middle school | 5 (3.6) | 9 (6.1) |  |
| Senior High school | 14 (10.1) | 9 (6.1) |  |
| College or above | 119 (86.2) | 130 (87.8) |  |
| Mother job, n (%) ^c^ |  |  | 0.309 |
| Employees of state-owned enterprises and public institutions | 40 (29.0) | 32 (21.6) |  |
| Employees of foreign-funded, private and enterprises | 54 (39.1) | 63 (42.6) |  |
| Other (farmers, soldiers, freelancers) | 30 (21.7) | 42 (28.4) |  |
| Unemployed | 14 (10.1) | 11 (7.4) |  |

Abbreviations: SD, 95% confidential interval.

^a^ For children’s age and parents’ education, the study analyzed by Wilcoxon rank sum test between intervention and control group.

^b^ For parents’ age, the study analyzed by two independent t test between intervention and control group.

^c^ For parents’ occupation, the study analyzed by Chi-square test between intervention and control group.

Table S2 The socio-demographic characters comparing intervention with control group for Jiading

| Variable | Intervention group  n=74 | Control group  n=64 | *P* value |
| --- | --- | --- | --- |
| Children age, n (%) ^a^ |  |  | 0.813 |
| 0 | 24 (32.4) | 20 (31.3) |  |
| 1 | 19 (25.7) | 19 (29.7) |  |
| 2 | 19 (25.7) | 10 (15.6) |  |
| 3 | 12 (16.2) | 15 (23.4) |  |
| Father age, y, mean (SD) ^b^ | 33.5 (4.7) | 34.1 (5.5) | 0.463 |
| Father education, n (%) ^a^ |  |  | 0.370 |
| Middle school | 0 (0) | 2 (3.1) |  |
| Senior High school | 6 (8.1) | 6 (9.38) |  |
| College or above | 68 (91.9) | 56 (87.5) |  |
| Father job, n (%) ^c^ |  |  | 0.309 |
| Employees of state-owned enterprises and public institutions | 26 (35.1) | 22 (34.4) |  |
| Employees of foreign-funded, private and enterprises | 32 (43.2) | 34 (53.1) |  |
| Other (farmers, soldiers, freelancers) | 16 (21.6) | 8 (12.5) |  |
| Unemployed | 0 (0) | 0 (0) |  |
| Mother age, y, mean (SD) ^b^ | 31.5 (3.7) | 32.2 (4.8) | 0.337 |
| Mother education, n (%) ^a^ |  |  | 0.588 |
| Middle school | 3 (4.1) | 2 (3.1) |  |
| Senior High school | 6 (8.1) | 8 (12.5) |  |
| College or above | 65 (87.8) | 54 (84.4) |  |
| Mother job, n (%) ^c^ |  |  | 0.615 |
| Employees of state-owned enterprises and public institutions | 21 (28.4) | 19 (29.7) |  |
| Employees of foreign-funded, private and enterprises | 26 (35.1) | 28 (43.8) |  |
| Other (farmers, soldiers, freelancers) | 18 (24.3) | 12 (18.8) |  |
| Unemployed | 9 (12.2) | 5 (7.8) |  |

Abbreviations: SD, 95% confidential interval.

^a^ For children’s age and parents’ education, the study analyzed by Wilcoxon rank sum test between intervention and control group.

^b^ For parents’ age, the study analyzed by two independent t test between intervention and control group.

^c^ For parents’ occupation, the study analyzed by Chi-square test between intervention and control group.

Table S3 The socio-demographic characters comparing intervention with control group for Juyuan

| Variable | Intervention group  n=78 | Control group  n=70 | *P* value |
| --- | --- | --- | --- |
| Children age, n (%) ^a^ |  |  | 0.823 |
| 0 | 28 (35.9) | 29 (41.4) |  |
| 1 | 27 (34.6) | 19 (27.1) |  |
| 2 | 13 (16.7) | 11 (15.7) |  |
| 3 | 10 (12.8) | 11 (15.7) |  |
| Father age, y, mean (SD) ^b^ | 32.9 (4.4) | 33.7 (4.1) | 0.218 |
| Father education, n (%) ^a^ |  |  | 0.781 |
| Middle school | 3 (3.9) | 3 (4.3) |  |
| Senior High school | 6 (7.7) | 4 (5.7) |  |
| College or above | 69 (88.5) | 63 (90.00) |  |
| Father job, n (%) ^c^ |  |  | 0.156 |
| Employees of state-owned enterprises and public institutions | 21 (26.92) | 28 (40.0) |  |
| Employees of foreign-funded, private and enterprises | 37 (47.44) | 31 (44.3) |  |
| Other (farmers, soldiers, freelancers) | 20 (25.64) | 11 (15.7) |  |
| Unemployed | 0 (0.0) | 0 (0.0) |  |
| Mother age, y, mean (SD) ^b^ | 31.2 (4.0) | 32.3 (4.0) | 0.093 |
| Mother education, n (%) ^a^ |  |  | 0.467 |
| Middle school | 5 (6.4) | 4 (5.7) |  |
| Senior High school | 6 (7.7) | 3 (4.3) |  |
| College or above | 67 (85.9) | 63 (90.0) |  |
| Mother job, n (%) ^c^ |  |  | 0.349 |
| Employees of state-owned enterprises and public institutions | 14 (18.0) | 18 (25.7) |  |
| Employees of foreign-funded, private and enterprises | 31 (39.7) | 32 (45.7) |  |
| Other (farmers, soldiers, freelancers) | 26 (33.3) | 16 (22.9) |  |
| Unemployed | 7 (9.0) | 4 (5.7) |  |

Abbreviations: SD, 95% confidential interval.

^a^ For children’s age and parents’ education, the study analyzed by Wilcoxon rank sum test between intervention and control group.

^b^ For parents’ age, the study analyzed by two independent t test between intervention and control group.

^c^ For parents’ occupation, the study analyzed by Chi-square test between intervention and control group.

Table S4 The socio-demographic characters of intervention groups for Jiading and Juyuan

| Variable | Intervention group  n=74 | Control group  n=78 | *P* value |
| --- | --- | --- | --- |
| Children age, n (%) ^a^ |  |  | 0.277 |
| 0 | 24 (32.4) | 28 (35.9) |  |
| 1 | 19 (25.7) | 27 (34.6) |  |
| 2 | 19 (25.7) | 13 (16.7) |  |
| 3 | 12 (16.22) | 10 (12.8) |  |
| Father age, y, mean (SD) ^b^ | 33.5 (4.7) | 32.9 (4.4) | 0.395 |
| Father education, n (%) ^a^ |  |  | 0.441 |
| Middle school | 0 (0.0) | 3 (3.9) |  |
| Senior High school | 6 (8.1) | 6 (7.7) |  |
| College or above | 68 (91.9) | 69 (88.5) |  |
| Father job, n (%) ^c^ |  |  | 0.539 |
| Employees of state-owned enterprises and public institutions | 26 () | 21 (26.9) |  |
| Employees of foreign-funded, private and enterprises | 32 () | 37 (47.4) |  |
| Other (farmers, soldiers, freelancers) | 16 () | 20 (25.6) |  |
| Unemployed | 0 (0.0) | 0 (0.0) |  |
| Mother age, y, mean (SD) ^b^ | 31.5 (3.7) | 31.2 (4.0) | 0.685 |
| Mother education, n (%) ^a^ |  |  | 0.697 |
| Middle school | 3 (4.1) | 5 (6.4) |  |
| Senior High school | 6 (8.1) | 6 (7.7) |  |
| College or above | 65 (87.8) | 67 (85.9) |  |
| Mother job, n (%) ^c^ |  |  | 0.329 |
| Employees of state-owned enterprises and public institutions | 21 (28.4) | 14 (18.0) |  |
| Employees of foreign-funded, private and enterprises | 26 (35.1) | 31 (39.7) |  |
| Other (farmers, soldiers, freelancers) | 18 (24.3) | 26 (33.3) |  |
| Unemployed | 9 (12.2) | 7 (9.0) |  |

Abbreviations: SD, 95% confidential interval.

^a^ For children’s age and parents’ education, the study analyzed by Wilcoxon rank sum test between intervention and control group.

^b^ For parents’ age, the study analyzed by two independent t test between intervention and control group.

^c^ For parents’ occupation, the study analyzed by Chi-square test between intervention and control group.

Table S5 The socio-demographic characters of control groups for Jiading and Juyuan

| Variable | Intervention group  n=64 | Control group  n=70 | *P* value |
| --- | --- | --- | --- |
| Children age, n (%) ^a^ |  |  | 0.187 |
| 0 | 20 (31.3) | 29 (41.4) |  |
| 1 | 19 (29.7) | 19 (27.1) |  |
| 2 | 10 (15.6) | 11 (15.7) |  |
| 3 | 15 (23.4) | 11 (15.7) |  |
| Father age, y, mean (SD) ^b^ | 34.1 (5.5) | 33.7 (4.1) | 0.637 |
| Father education, n (%) ^a^ |  |  | 0.678 |
| Middle school | 2 (3.1) | 3 (4.3) |  |
| Senior High school | 6 (9.38) | 4 (5.7) |  |
| College or above | 56 (87.5) | 63 (90.00) |  |
| Father job, n (%) ^c^ |  |  | 0.587 |
| Employees of state-owned enterprises and public institutions | 22 (34.4) | 28 (40.0) |  |
| Employees of foreign-funded, private and enterprises | 34 (53.1) | 31 (44.3) |  |
| Other (farmers, soldiers, freelancers) | 8 (12.5) | 11 (15.7) |  |
| Unemployed | 0 (0) | 0 (0.0) |  |
| Mother age, y, mean (SD) ^b^ | 32.2 (4.8) | 32.3 (4.0) | 0.822 |
| Mother education, n (%) ^a^ |  |  | 0.384 |
| Middle school | 2 (3.1) | 4 (5.7) |  |
| Senior High school | 8 (12.5) | 3 (4.3) |  |
| College or above | 54 (84.4) | 63 (90.0) |  |
| Mother job, n (%) ^c^ |  |  | 0.871 |
| Employees of state-owned enterprises and public institutions | 19 (29.7) | 18 (25.7) |  |
| Employees of foreign-funded, private and enterprises | 28 (43.8) | 32 (45.7) |  |
| Other (farmers, soldiers, freelancers) | 12 (18.8) | 16 (22.9) |  |
| Unemployed | 5 (7.8) | 4 (5.7) |  |

Abbreviations: SD, 95% confidential interval.

^a^ For children’s age and parents’ education, the study analyzed by Wilcoxon rank sum test between intervention and control group.

^b^ For parents’ age, the study analyzed by two independent t test between intervention and control group.

^c^ For parents’ occupation, the study analyzed by Chi-square test between intervention and control group.


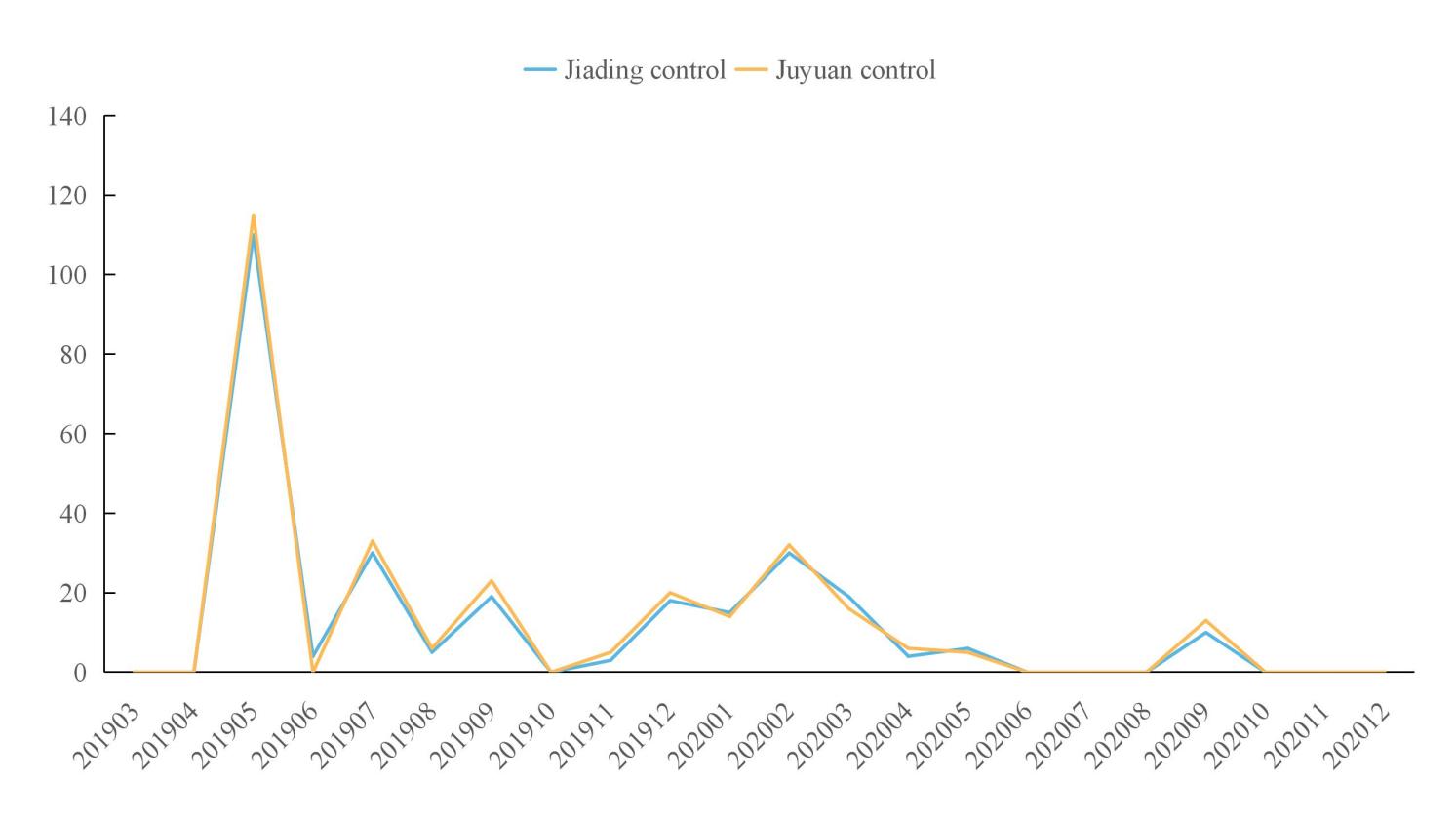


Supplementary Material Figure S1 The time-series plot of control group chatting records for Jiading and Juyuan


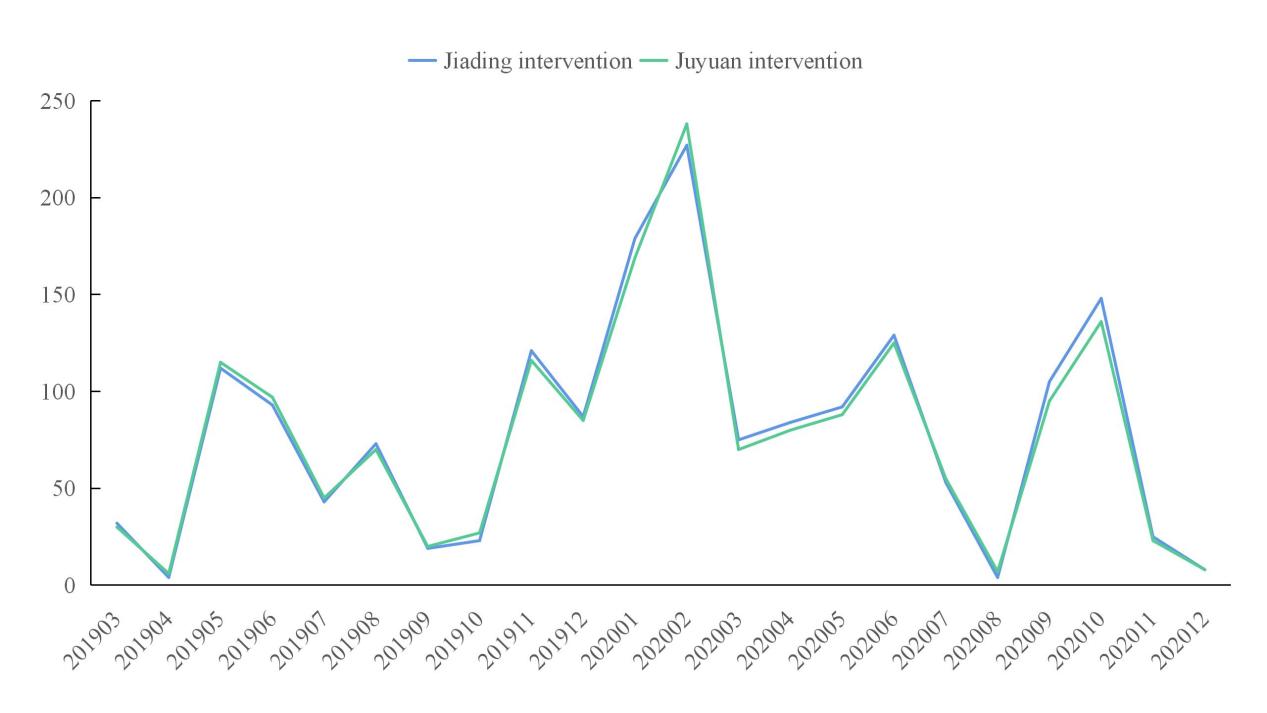


Supplementary Material Figure S2 The time-series plot of intervention group chatting records for Jiading and Juyuan


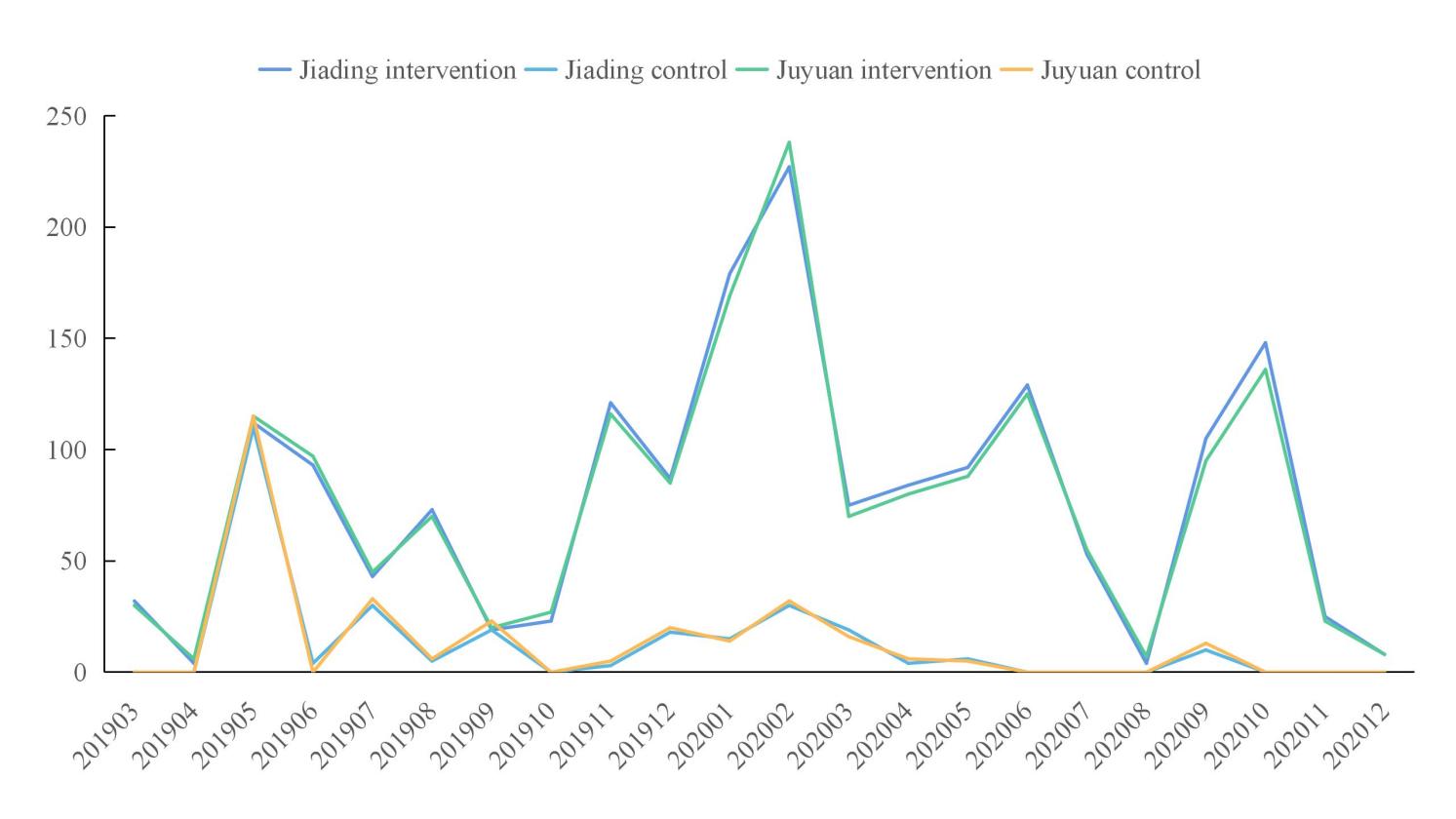


Supplementary Material Figure S3 The time-series plot of intervention group and control group chatting records for Jiading and Juyuan

Table S6 The number of intervention group and control group chatting records for Jiading and Juyuan

| Time | Jiading intervention | Jiading control | Juyuan intervention | Juyuan control |
| --- | --- | --- | --- | --- |
| 201903 | 32 | 0 | 30 | 0 |
| 201904 | 4 | 0 | 6 | 0 |
| 201905 | 112 | 110 | 115 | 115 |
| 201906 | 93 | 4 | 97 | 4 |
| 201907 | 43 | 30 | 45 | 33 |
| 201908 | 73 | 5 | 70 | 6 |
| 201909 | 19 | 19 | 20 | 23 |
| 201910 | 23 | 0 | 27 | 0 |
| 201911 | 121 | 3 | 116 | 5 |
| 201912 | 87 | 18 | 85 | 20 |
| 202001 | 179 | 15 | 169 | 14 |
| 202002 | 227 | 30 | 238 | 32 |
| 202003 | 75 | 19 | 70 | 16 |
| 202004 | 84 | 4 | 80 | 6 |
| 202005 | 92 | 6 | 88 | 5 |
| 202006 | 129 | 0 | 125 | 0 |
| 202007 | 53 | 0 | 55 | 0 |
| 202008 | 4 | 0 | 7 | 0 |
| 202009 | 105 | 10 | 95 | 13 |
| 202010 | 148 | 0 | 136 | 0 |
| 202011 | 25 | 0 | 23 | 0 |
| 202012 | 8 | 0 | 8 | 0 |

Table S7 The proportion chatting frequencies of intervention group and control group in Jaiding and Juyuan

| Chatting frequencies | Intervention group [n (%)] | | |  | Control group [n (%)] | | |
| --- | --- | --- | --- | --- | --- | --- | --- |
|  | Jiading  (n=74) | Juyuan  (n=78) | P value |  | Jiading  (n=64) | Juyuan  (n=70) | P value |
|  |  |  | 0.816 |  |  |  | 0.377 |
| 0 | 20 (27.0) | 24 (30.8) |  |  | 44 (68.8) | 43 (61.4) |  |
| 1-30 | 44 (59.5) | 38 (48.7) |  |  | 20 (31.2) | 27 (38.6) |  |
| 31-60 | 6 (9.4) | 9 (11.5) |  |  | 0 (0.0) | 0 (0.0) |  |
| 61- | 4 (6.2) | 7 (8.9) |  |  | 0 (0.0) | 0 (0.0) |  |

# The socio-demographic characteristics comparison of participants who withdrawn and completed in Jiading and Juyuan

Table S8 The socio-demographic characters comparing withdrawn with completed in Jiading and Juyuan

| Variable | Withdrawn  n=89 | Completed  n=276 | *P* value |
| --- | --- | --- | --- |
| Children age, n (%) ^a^ |  |  | 0.404 |
| 0 | 19 (24.1) | 101 (35.3) |  |
| 1 | 32 (40.5) | 84 (29.4) |  |
| 2 | 18 (22.8) | 53 (18.5) |  |
| 3 | 10 (12.7) | 48 (16.8) |  |
| Father age, y, mean (SD) ^b^ | 32.7 (4.6) | 33.5 (4.7) | 0.194 |
| Father education, n (%) ^a^ |  |  | 0.803 |
| Middle school | 3 (3.8) | 8 (2.8) |  |
| Senior High school | 6 (7.6) | 22 (7.7) |  |
| College or above | 70 (88.6) | 256 (89.5) |  |
| Father job, n (%) ^c^ |  |  | 0.106 |
| Employees of state-owned enterprises and public institutions | 17 (21.5) | 97 (33.9) |  |
| Employees of foreign-funded, private and enterprises | 43 (54.4) | 134 (46.9) |  |
| Other (farmers, soldiers, freelancers) | 19 (24.1) | 55 (19.2) |  |
| Unemployed | 0 (0.0) | 0 (0.0) |  |
| Mother age, y, mean (SD) ^b^ | 31.3 (3.8) | 31.8 (4.1) | 0.341 |
| Mother education, n (%) ^a^ |  |  | 0.121 |
| Middle school | 4 (5.1) | 14 (4.9) |  |
| Senior High school | 12 (15.2) | 23 (8.0) |  |
| College or above | 63 (79.8) | 249 (87.1) |  |
| Mother job, n (%) ^c^ |  |  | 0.680 |
| Employees of state-owned enterprises and public institutions | 15 (19.0) | 72 (25.2) |  |
| Employees of foreign-funded, private and enterprises | 37 (46.8) | 117 (40.9) |  |
| Other (farmers, soldiers, freelancers) | 20 (25.3) | 72 (25.2) |  |
| Unemployed | 7 (8.9) | 25 (8.7) |  |

Abbreviations: SD, 95% confidential interval.

^a^ For children’s age and parents’ education, the study analyzed by Wilcoxon rank sum test between intervention and control group.

^b^ For parents’ age, the study analyzed by two independent t test between intervention and control group.

^c^ For parents’ occupation, the study analyzed by Chi-square test between intervention and control group

Table S9 The socio-demographic characters comparing withdrawn with completed in Jiading

| Variable | Withdrawn  n=37 | Completed  n=138 | *P* value |
| --- | --- | --- | --- |
| Children age, n (%) ^a^ |  |  | 0.619 |
| 0 | 11 (29.7) | 44 (31.9) |  |
| 1 | 14 (37.8) | 38 (27.5) |  |
| 2 | 7 (18.9) | 29 (21.0) |  |
| 3 | 5 (13.5) | 27 (19.6) |  |
| Father age, y, mean (SD) ^b^ | 33.2 (5.4) | 33.8 (5.1) | 0.553 |
| Father education, n (%) ^a^ |  |  | 0.490 |
| Middle school | 3 (8.1) | 2 (1.5) |  |
| Senior High school | 2 (5.4) | 12 (8.7) |  |
| College or above | 32 (86.5) | 124 (89.9) |  |
| Father job, n (%) ^c^ |  |  | 0.473 |
| Employees of state-owned enterprises and public institutions | 9 (24.3) | 48 (34.8) |  |
| Employees of foreign-funded, private and enterprises | 20 (54.1) | 66 (47.8) |  |
| Other (farmers, soldiers, freelancers) | 8 (21.6) | 24 (17.4) |  |
| Unemployed | 0 (0.0) | 0 (0.0) |  |
| Mother age, y, mean (SD) ^b^ | 31.9 (4.4) | 31.8 (4.2) | 0.917 |
| Mother education, n (%) ^a^ |  |  | 0.053 |
| Middle school | 3 (8.1) | 5 (3.6) |  |
| Senior High school | 7 (18.9) | 4 (10.1) |  |
| College or above | 27 (73.0) | 119 (86.2) |  |
| Mother job, n (%) ^c^ |  |  | 0.543 |
| Employees of state-owned enterprises and public institutions | 7 (18.9) | 40 (29.0) |  |
| Employees of foreign-funded, private and enterprises | 16 (43.2) | 54 (39.1) |  |
| Other (farmers, soldiers, freelancers) | 11 (29.7) | 30 (21.7) |  |
| Unemployed | 3 (8.1) | 14 (10.1) |  |

Abbreviations: SD, 95% confidential interval.

^a^ For children’s age and parents’ education, the study analyzed by Wilcoxon rank sum test between intervention and control group.

^b^ For parents’ age, the study analyzed by two independent t test between intervention and control group.

^c^ For parents’ occupation, the study analyzed by Chi-square test between intervention and control group

Table S10 The socio-demographic characters comparing withdrawn with completed in Juyuan

| Variable | Withdrawn  n=42 | Completed  n=148 | *P* value |
| --- | --- | --- | --- |
| Children age, n (%) ^a^ |  |  | 0.088 |
| 0 | 8 (19.1) | 57 (38.5) |  |
| 1 | 18 (42.9) | 46 (31.1) |  |
| 2 | 11 (26.2) | 24 (16.2) |  |
| 3 | 5 (11.9) | 21 (14.2) |  |
| Father age, y, mean (SD) ^b^ | 32.3 (3.8) | 33.3 (4.3) | 0.201 |
| Father education, n (%) ^a^ |  |  | 0.756 |
| Middle school | 0 (0.0) | 6 (4.1) |  |
| Senior High school | 4 (9.5) | 10 (6.8) |  |
| College or above | 38 (90.5) | 132 (89.2) |  |
| Father job, n (%) ^c^ |  |  | 0.213 |
| Employees of state-owned enterprises and public institutions | 8 (19.1) | 49 (33.1) |  |
| Employees of foreign-funded, private and enterprises | 23 (54.8) | 68 (46.0) |  |
| Other (farmers, soldiers, freelancers) | 11 (26.2) | 31 (21,0) |  |
| Unemployed | 0 (0.0) | 0 (0.0) |  |
| Mother age, y, mean (SD) ^b^ | 30.7 (3.2) | 31.7 (4.1) | 0.143 |
| Mother education, n (%) ^a^ |  |  | 0.791 |
| Middle school | 1 (2.4) | 9 (6.1) |  |
| Senior High school | 5 (11.9) | 9 (6.1) |  |
| College or above | 36 (85.7) | 130 (87.8) |  |
| Mother job, n (%) ^c^ |  |  | 0.733 |
| Employees of state-owned enterprises and public institutions | 8 (19.1) | 32 (21.6) |  |
| Employees of foreign-funded, private and enterprises | 21 (50.0) | 63 (42.6) |  |
| Other (farmers, soldiers, freelancers) | 9 (21.4) | 42 (28.4) |  |
| Unemployed | 4 (9.5) | 11 (7.4) |  |

Abbreviations: SD, 95% confidential interval.

^a^ For children’s age and parents’ education, the study analyzed by Wilcoxon rank sum test between intervention and control group.

^b^ For parents’ age, the study analyzed by two independent t test between intervention and control group.

^c^ For parents’ occupation, the study analyzed by Chi-square test between intervention and control group

# Study processing

Table S11 The classification and focus of the 30 articles

| Classification | Articles | Teaching effect index | |
| --- | --- | --- | --- |
|  |  | Skills | Awareness |
| Falls |  |  |  |
|  | How to prevent babies aged 0-3 from falling, here's the full answer! | ++ | ++ |
|  | Tips for preventing falls in children. | ++ | ++ |
|  | You can do more to prevent your baby from falling | ++ | ++ |
| Burns |  |  |  |
|  | Put cold water or hot water first when bathing your baby? Unexpectedly, the correct way is.... | +++ | +++ |
|  | Here's the correct way to treat children's burns, come and check. | +++ | + |
|  | When parents give the baby a bath, put hot water first easy to burn, the correct approach is...... | ++ | +++ |
|  | How does darling burn do? Here's the answer! | +++ | + |
|  | A moment of negligence breeds tragedy! What parents need to know to prevent children from burning. | + | +++ |
| Drowning |  |  |  |
|  | Water is a source of life, but it can also rob people of their lives: prevent drowning in children, keep this in mind! | ++ | +++ |
|  | Does drowning happen in your home? The result was...... | + | +++ |
|  | A safe and happy swimming guide for children aged 0-3. | +++ | +++ |
|  | Preventing children from drowning at home, what parents have to know? | ++ | +++ |
|  | Children drowning first aid knowledge get up ~ | +++ | +++ |
| Poisoning |  |  |  |
|  | "Keep it high, keep it away from children"--small lecture hall for children poisoning prevention | ++ | +++ |
|  | Tips for preventing poisoning in children. | +++ | +++ |
|  | Alert! Remember these tips to save your baby's life when he gets poisoned | +++ | +++ |
| Asphyxia |  |  |  |
|  | Babies always choke water/milk, what should parents do? Here are a few ways to help you solve it easily | +++ | +++ |
|  | Children are prevented from choking before the age of 1. | ++ | +++ |
|  | 10 “not do” parents should pay attention to prevent baby asphyxia | ++ | +++ |
|  | First aid advice: Is it correct to pat children directly on the back for foreign body asphyxia? | +++ | ++ |
|  | Is your home also a child asphyxia hotspot? Prevention of suffocation at home can not be ignored! | + | +++ |
| Other |  |  |  |
|  | When buying things for your baby, pay attention to these! | + | +++ |
|  | Should babies sleep with their parents? | ++ | +++ |
|  | Do you know? There are invisible killers in your home. | + | +++ |
|  | Real cases of injuries in children under one year of age, parents must pay attention. | + | +++ |
|  | Parents should be the “safe umbrella” suitable for children's age. | ++ | +++ |
|  | When taking care of children, your eyes must not leave the children, even for a minute! | ++ | +++ |
|  | Children happen ankle sprain, should rest or massage? Cold compress or hot compress? | +++ | ++ |
|  | Parents must know: children happened trauma how to deal with! | +++ | +++ |
|  | Unintentional injuries to children frequently occur in summer, parents need to be aware. | + | +++ |

Table S12 The screening criteria of interactions

| Ties type | The count of out point | The count of in point |
| --- | --- | --- |
| Ask and answer interaction |  |  |
| 1→1 | Asking questions automatically and count it once. If an information contain many questions, count it based on questions types. | Answering questions automatically and count it once. If an information contain many answers, count it based on questions types. |
| 1→n | Asking questions automatically and count it once. If an information contain many questions, count it based on questions types. | Counting based on the source of point in. Answering questions for one source and count it once If an information contain many answers, count it based on questions types. |
| n→1 | Counting based on the source of point out. Asking questions for one source and count it once If an information contain many answers, count it based on questions types. | Answering questions automatically and count it once. If an information contain many answers, count it based on questions types. |
| Education interaction |  |  |
| 1→1 | — | — |
| 1→n | Delivering health education articles or related childcare knowledge | Answering health education articles or related childcare knowledge |
| n→1 | — | — |

# The implication of study indicators and results

## Ego network

### Coreness

Table S13 The classification criteria of coreness

| Classification | Criteria |
| --- | --- |
| Core | Coreness>0.1 |
| Activity | 0.01<Coreness≤0.1 |
| Little activity | 0.001<Coreness≤0.01 |
| Silence | 0<Coreness≤0.001 |
| Alienation | Coreness=0 |

### Basic ego measures

In this study, the implication of ego network analysis means that individuals are extracted from the whole network and their social network get measured. Five indicators are measured and shown, including size of ego network, number of directed ties, density, two-step reach, and reach efficiency. The measurement methods and implications of them are as follows (about parents A). The results are shown in Table S8~S9.

#### Size of ego network

This indicator demonstrates the number of nodes which linked parent A directly instead of going through other nodes. This number also includes parent A. The bigger size of ego network means that parent A plays more important role in the online-social-network-based parental health education.

#### Number of directed ties

This indicator demonstrates the number of links in the ego network of parent A. This indicator reflects the interaction relationship of network members. The bigger number of directed ties means that parent A’s ego network more strength, because network members usually help mutually, and parent A is more cohesive.

#### Density

This indicator demonstrates the status of ego network, which calculated by the number of links in actually (m) divided by the number of links in possibly (n). The smaller density means that this ego network needs community childcare doctors’ help much more.

#### Two-step reach

This indicator demonstrates the nodes’ proportion of the information delivery distance equal or less than 2 in the ego network. We assume three parents (parent A, parent B, and parent C) included in an ego network. If parent A wants to deliver information to parent C, he can deliver either via parent B or to parent C directly. This is the meaning of two-step reach. This indicator reflects the ease of child-unintentional injury-related information delivery through the ego network. The bigger two-step reach means the easier information delivery.

#### Reach efficiency

This indicator, as a standard indicator, is calculated by “two-step reach” divided by “size of ego network”. The bigger reach efficiency means that parent A’s neighbors (other parents in his ego network) can complement the lack links in parent A’s ego network better and parent A can get broader communication with other parents and community childcare doctors.

Table S14 The ego network measures of control group

| Number | ID | Speeches | Days | Coreness | Size | Tie | Density | 2StepR | ReachE | Type |
| --- | --- | --- | --- | --- | --- | --- | --- | --- | --- | --- |
| 1 | 1006 | 9 | 6 | 0.126 | 1 | 0 | 0.00 | 26.15 | 100.00 | core |
| 2 | 1012 | 0 | 0 | 0.000 | 0 | 0 | 0.00 | 0.00 | 0.00 | alienation |
| 3 | 1013 | 0 | 0 | 0.000 | 0 | 0 | 0.00 | 0.00 | 0.00 | alienation |
| 4 | 1016 | 0 | 0 | 0.000 | 0 | 0 | 0.00 | 0.00 | 0.00 | alienation |
| 5 | 1023 | 0 | 0 | 0.000 | 0 | 0 | 0.00 | 0.00 | 0.00 | alienation |
| 6 | 1025 | 0 | 0 | 0.000 | 0 | 0 | 0.00 | 0.00 | 0.00 | alienation |
| 7 | 1027 | 0 | 0 | 0.000 | 0 | 0 | 0.00 | 0.00 | 0.00 | alienation |
| 8 | 1029 | 0 | 0 | 0.000 | 0 | 0 | 0.00 | 0.00 | 0.00 | alienation |
| 9 | 1036 | 0 | 0 | 0.000 | 0 | 0 | 0.00 | 0.00 | 0.00 | alienation |
| 10 | 1038 | 0 | 0 | 0.000 | 0 | 0 | 0.00 | 0.00 | 0.00 | alienation |
| 11 | 1039 | 0 | 0 | 0.000 | 0 | 0 | 0.00 | 0.00 | 0.00 | alienation |
| 12 | 1040 | 2 | 1 | 0.126 | 1 | 0 | 0.00 | 26.15 | 100.00 | core |
| 13 | 1041 | 0 | 0 | 0.000 | 0 | 0 | 0.00 | 0.00 | 0.00 | alienation |
| 14 | 1049 | 0 | 0 | 0.000 | 0 | 0 | 0.00 | 0.00 | 0.00 | alienation |
| 15 | 1051 | 0 | 0 | 0.000 | 0 | 0 | 0.00 | 0.00 | 0.00 | alienation |
| 16 | 1055 | 0 | 0 | 0.000 | 0 | 0 | 0.00 | 0.00 | 0.00 | alienation |
| 17 | 1057 | 0 | 0 | 0.000 | 0 | 0 | 0.00 | 0.00 | 0.00 | alienation |
| 18 | 1059 | 0 | 0 | 0.000 | 0 | 0 | 0.00 | 0.00 | 0.00 | alienation |
| 19 | 1062 | 0 | 0 | 0.000 | 2 | 2 | 100.00 | 26.15 | 85.00 | alienation |
| 20 | 1065 | 0 | 0 | 0.000 | 0 | 0 | 0.00 | 0.00 | 0.00 | alienation |
| 21 | 1069 | 0 | 0 | 0.000 | 0 | 0 | 0.00 | 0.00 | 0.00 | alienation |
| 22 | 1078 | 0 | 0 | 0.000 | 0 | 0 | 0.00 | 0.00 | 0.00 | alienation |
| 23 | 1079 | 24 | 1 | 0.173 | 2 | 2 | 100.00 | 27.69 | 78.26 | core |
| 24 | 1083 | 0 | 0 | 0.000 | 0 | 0 | 0.00 | 0.00 | 0.00 | alienation |
| 25 | 1090 | 0 | 0 | 0.000 | 0 | 0 | 0.00 | 0.00 | 0.00 | alienation |
| 26 | 1093 | 2 | 2 | 0.012 | 1 | 0 | 0.00 | 6.15 | 100.00 | activity |
| 27 | 1094 | 0 | 0 | 0.000 | 0 | 0 | 0.00 | 0.00 | 0.00 | alienation |
| 28 | 1096 | 0 | 0 | 0.000 | 0 | 0 | 0.00 | 0.00 | 0.00 | alienation |
| 29 | 1099 | 0 | 0 | 0.000 | 0 | 0 | 0.00 | 0.00 | 0.00 | alienation |
| 30 | 1101 | 0 | 0 | 0.000 | 0 | 0 | 0.00 | 0.00 | 0.00 | alienation |
| 31 | 1103 | 0 | 0 | 0.000 | 0 | 0 | 0.00 | 0.00 | 0.00 | alienation |
| 32 | 1104 | 33 | 4 | 0.269 | 6 | 8 | 26.67 | 32.31 | 65.63 | core |
| 33 | 1105 | 0 | 0 | 0.000 | 0 | 0 | 0.00 | 0.00 | 0.00 | alienation |
| 34 | 1106 | 1 | 1 | 0.000 | 1 | 0 | 0.00 | 6.15 | 100.00 | alienation |
| 35 | 1107 | 0 | 0 | 0.000 | 0 | 0 | 0.00 | 0.00 | 0.00 | alienation |
| 36 | 1108 | 2 | 2 | 0.063 | 2 | 0 | 0.00 | 9.23 | 85.71 | activity |
| 37 | 1111 | 10 | 3 | 0.173 | 2 | 2 | 100.00 | 27.69 | 78.26 | core |
| 38 | 1123 | 0 | 0 | 0.000 | 0 | 0 | 0.00 | 0.00 | 0.00 | alienation |
| 39 | 1128 | 17 | 5 | 0.246 | 5 | 7 | 35.00 | 29.23 | 57.58 | core |
| 40 | 1130 | 0 | 0 | 0.000 | 0 | 0 | 0.00 | 0.00 | 0.00 | alienation |
| 41 | 1140 | 0 | 0 | 0.000 | 0 | 0 | 0.00 | 0.00 | 0.00 | alienation |
| 42 | 1143 | 0 | 0 | 0.000 | 0 | 0 | 0.00 | 0.00 | 0.00 | alienation |
| 43 | 1145 | 30 | 13 | 0.166 | 4 | 4 | 33.33 | 32.31 | 70.00 | core |
| 44 | 1151 | 0 | 0 | 0.000 | 0 | 0 | 0.00 | 0.00 | 0.00 | alienation |
| 45 | 1160 | 0 | 0 | 0.000 | 0 | 0 | 0.00 | 0.00 | 0.00 | alienation |
| 46 | 1167 | 0 | 0 | 0.000 | 0 | 0 | 0.00 | 0.00 | 0.00 | alienation |
| 47 | 1169 | 7 | 2 | 0.173 | 2 | 2 | 100.00 | 27.69 | 78.26 | core |
| 48 | 1170 | 0 | 0 | 0.000 | 0 | 0 | 0.00 | 0.00 | 0.00 | alienation |
| 49 | 1171 | 2 | 1 | 0.126 | 1 | 0 | 0.00 | 26.15 | 100.00 | core |
| 50 | 1173 | 0 | 0 | 0.000 | 0 | 0 | 0.00 | 0.00 | 0.00 | alienation |
| 51 | 1180 | 0 | 0 | 0.000 | 0 | 0 | 0.00 | 0.00 | 0.00 | alienation |
| 52 | 1190 | 0 | 0 | 0.000 | 0 | 0 | 0.00 | 0.00 | 0.00 | alienation |
| 53 | 1194 | 2 | 2 | 0.126 | 1 | 0 | 0.00 | 26.15 | 100.00 | core |
| 54 | 1198 | 0 | 0 | 0.000 | 0 | 0 | 0.00 | 0.00 | 0.00 | alienation |
| 55 | 1200 | 19 | 5 | 0.152 | 3 | 2 | 33.33 | 26.15 | 73.91 | core |
| 56 | 1201 | 3 | 1 | 0.136 | 2 | 0 | 0.00 | 27.69 | 94.74 | core |
| 57 | 1203 | 2 | 2 | 0.126 | 1 | 0 | 0.00 | 26.15 | 100.00 | core |
| 58 | 1212 | 0 | 0 | 0.000 | 0 | 0 | 0.00 | 0.00 | 0.00 | alienation |
| 59 | 1223 | 0 | 0 | 0.000 | 0 | 0 | 0.00 | 0.00 | 0.00 | alienation |
| 60 | 1224 | 8 | 7 | 0.074 | 4 | 0 | 0.00 | 15.38 | 83.33 | activity |
| 61 | 1230 | 0 | 0 | 0.000 | 0 | 0 | 0.00 | 0.00 | 0.00 | alienation |
| 62 | 1248 | 7 | 1 | 0.126 | 1 | 0 | 0.00 | 26.15 | 100.00 | core |
| 63 | 1250 | 0 | 0 | 0.000 | 0 | 0 | 0.00 | 0.00 | 0.00 | alienation |
| 64 | 1257 | 2 | 1 | 0.126 | 1 | 0 | 0.00 | 26.15 | 100.00 | core |
| 65 | 8888 | 3 | 2 | 0.154 | 4 | 7 | 58.33 | 29.23 | 65.52 | core |
| 66 | 9999 | 88 | 21 | 0.751 | 17 | 14 | 5.15 | 29.23 | 48.72 | core |

Table S15 The ego network measures of intervention group

| Number | ID | Speeches | Days | Coreness | Size | Tie | Density | 2StepR | ReachE | Type |
| --- | --- | --- | --- | --- | --- | --- | --- | --- | --- | --- |
| 1 | 1003 | 2 | 1 | 0.012 | 2 | 0 | 0.00 | 12.00 | 100.00 | activity |
| 2 | 1004 | 51 | 28 | 0.241 | 13 | 56 | 35.90 | 64.00 | 30.19 | core |
| 3 | 1010 | 0 | 0 | 0.000 | 0 | 0 | 0.00 | 0.00 | 0.00 | alienation |
| 4 | 1011 | 0 | 0 | 0.000 | 0 | 0 | 0.00 | 0.00 | 0.00 | alienation |
| 5 | 1015 | 8 | 3 | 0.053 | 1 | 0 | 0.00 | 58.67 | 100.00 | activity |
| 6 | 1017 | 4 | 2 | 0.059 | 2 | 0 | 0.00 | 64.00 | 94.12 | activity |
| 7 | 1021 | 0 | 0 | 0.000 | 1 | 0 | 0.00 | 58.67 | 100.00 | alienation |
| 8 | 1022 | 4 | 1 | 0.094 | 3 | 6 | 100.00 | 61.33 | 68.66 | activity |
| 9 | 1024 | 9 | 2 | 0.078 | 2 | 2 | 100.00 | 61.33 | 73.02 | activity |
| 10 | 1031 | 0 | 0 | 0.000 | 0 | 0 | 0.00 | 0.00 | 0.00 | alienation |
| 11 | 1033 | 0 | 0 | 0.000 | 0 | 0 | 0.00 | 0.00 | 0.00 | alienation |
| 12 | 1034 | 0 | 0 | 0.000 | 0 | 0 | 0.00 | 0.00 | 0.00 | alienation |
| 13 | 1035 | 0 | 0 | 0.000 | 0 | 0 | 0.00 | 0.00 | 0.00 | alienation |
| 14 | 1037 | 0 | 0 | 0.000 | 0 | 0 | 0.00 | 0.00 | 0.00 | alienation |
| 15 | 1042 | 0 | 0 | 0.000 | 0 | 0 | 0.00 | 0.00 | 0.00 | alienation |
| 16 | 1044 | 5 | 1 | 0.078 | 2 | 2 | 100.00 | 61.33 | 77.97 | activity |
| 17 | 1045 | 0 | 0 | 0.000 | 0 | 0 | 0.00 | 0.00 | 0.00 | alienation |
| 18 | 1046 | 13 | 4 | 0.071 | 5 | 11 | 55.00 | 61.33 | 59.74 | activity |
| 19 | 1054 | 27 | 7 | 0.053 | 1 | 0 | 0.00 | 58.67 | 100.00 | activity |
| 20 | 1056 | 24 | 9 | 0.149 | 10 | 49 | 54.44 | 61.33 | 33.09 | core |
| 21 | 1058 | 43 | 5 | 0.143 | 11 | 61 | 55.45 | 62.67 | 29.94 | core |
| 22 | 1060 | 0 | 0 | 0.000 | 0 | 0 | 0.00 | 0.00 | 0.00 | alienation |
| 23 | 1063 | 3 | 2 | 0.053 | 1 | 0 | 0.00 | 58.67 | 100.00 | activity |
| 24 | 1072 | 0 | 0 | 0.000 | 0 | 0 | 0.00 | 0.00 | 0.00 | alienation |
| 25 | 1074 | 157 | 38 | 0.278 | 17 | 95 | 34.93 | 64.00 | 24.62 | core |
| 26 | 1075 | 0 | 0 | 0.000 | 0 | 0 | 0.00 | 0.00 | 0.00 | alienation |
| 27 | 1077 | 4 | 1 | 0.053 | 1 | 0 | 0.00 | 58.67 | 100.00 | activity |
| 28 | 1080 | 44 | 7 | 0.137 | 9 | 48 | 66.67 | 61.33 | 31.72 | core |
| 29 | 1085 | 15 | 3 | 0.062 | 3 | 4 | 66.67 | 58.67 | 73.33 | activity |
| 30 | 1087 | 0 | 0 | 0.000 | 0 | 0 | 0.00 | 0.00 | 0.00 | alienation |
| 31 | 1089 | 0 | 0 | 0.000 | 0 | 0 | 0.00 | 0.00 | 0.00 | alienation |
| 32 | 1091 | 0 | 0 | 0.000 | 0 | 0 | 0.00 | 0.00 | 0.00 | alienation |
| 33 | 1095 | 17 | 3 | 0.092 | 5 | 13 | 65.00 | 62.67 | 48.96 | activity |
| 34 | 1100 | 0 | 0 | 0.000 | 2 | 0 | 0.00 | 14.67 | 91.67 | alienation |
| 35 | 1102 | 0 | 0 | 0.000 | 0 | 0 | 0.00 | 0.00 | 0.00 | alienation |
| 36 | 1109 | 16 | 10 | 0.174 | 8 | 35 | 62.50 | 61.33 | 38.33 | core |
| 37 | 1113 | 8 | 1 | 0.053 | 1 | 0 | 0.00 | 58.67 | 100.00 | activity |
| 38 | 1116 | 7 | 2 | 0.059 | 2 | 1 | 50.00 | 58.67 | 89.80 | activity |
| 39 | 1119 | 132 | 41 | 0.273 | 19 | 95 | 27.78 | 64.00 | 25.13 | core |
| 40 | 1129 | 7 | 2 | 0.053 | 1 | 0 | 0.00 | 58.67 | 100.00 | activity |
| 41 | 1132 | 20 | 6 | 0.091 | 5 | 13 | 65.00 | 62.67 | 47.96 | activity |
| 42 | 1136 | 10 | 3 | 0.053 | 2 | 2 | 100.00 | 58.67 | 77.19 | activity |
| 43 | 1139 | 0 | 0 | 0.000 | 0 | 0 | 0.00 | 0.00 | 0.00 | alienation |
| 44 | 1146 | 38 | 17 | 0.156 | 12 | 45 | 34.09 | 64.00 | 33.33 | core |
| 45 | 1147 | 23 | 5 | 0.075 | 3 | 4 | 66.67 | 58.67 | 64.71 | activity |
| 46 | 1149 | 4 | 2 | 0.053 | 1 | 0 | 0.00 | 58.67 | 100.00 | activity |
| 47 | 1154 | 2 | 1 | 0.053 | 1 | 0 | 0.00 | 58.67 | 100.00 | activity |
| 48 | 1157 | 0 | 0 | 0.000 | 0 | 0 | 0.00 | 0.00 | 0.00 | alienation |
| 49 | 1161 | 3 | 2 | 0.075 | 2 | 2 | 100.00 | 58.67 | 77.19 | activity |
| 50 | 1174 | 15 | 6 | 0.075 | 7 | 19 | 45.24 | 37.33 | 36.84 | activity |
| 51 | 1189 | 32 | 8 | 0.133 | 8 | 12 | 21.43 | 61.33 | 48.94 | core |
| 52 | 1191 | 3 | 2 | 0.006 | 1 | 0 | 0.00 | 9.33 | 100.00 | little activity |
| 53 | 1193 | 2 | 2 | 0.053 | 2 | 0 | 0.00 | 64.00 | 94.12 | activity |
| 54 | 1196 | 68 | 20 | 0.275 | 15 | 82 | 39.05 | 65.33 | 26.78 | core |
| 55 | 1197 | 0 | 0 | 0.000 | 0 | 0 | 0.00 | 0.00 | 0.00 | alienation |
| 56 | 1199 | 0 | 0 | 0.000 | 0 | 0 | 0.00 | 0.00 | 0.00 | alienation |
| 57 | 1202 | 24 | 13 | 0.098 | 5 | 14 | 70.00 | 61.33 | 48.94 | activity |
| 58 | 1204 | 3 | 1 | 0.014 | 1 | 0 | 0.00 | 16.00 | 100.00 | activity |
| 59 | 1209 | 0 | 0 | 0.000 | 0 | 0 | 0.00 | 0.00 | 0.00 | alienation |
| 60 | 1215 | 8 | 4 | 0.053 | 1 | 0 | 0.00 | 58.67 | 100.00 | activity |
| 61 | 1218 | 26 | 13 | 0.164 | 7 | 28 | 66.67 | 62.67 | 38.52 | core |
| 62 | 1220 | 0 | 0 | 0.000 | 0 | 0 | 0.00 | 0.00 | 0.00 | alienation |
| 63 | 1222 | 60 | 17 | 0.167 | 11 | 41 | 37.27 | 64.00 | 33.57 | core |
| 64 | 1225 | 0 | 0 | 0.000 | 1 | 0 | 0.00 | 2.67 | 100.00 | alienation |
| 65 | 1226 | 0 | 0 | 0.000 | 0 | 0 | 0.00 | 0.00 | 0.00 | alienation |
| 66 | 1227 | 0 | 0 | 0.000 | 0 | 0 | 0.00 | 0.00 | 0.00 | alienation |
| 67 | 1229 | 7 | 5 | 0.053 | 1 | 0 | 0.00 | 58.67 | 100.00 | activity |
| 68 | 1234 | 3 | 2 | 0.053 | 1 | 0 | 0.00 | 58.67 | 100.00 | activity |
| 69 | 1239 | 17 | 3 | 0.093 | 5 | 10 | 50.00 | 64.00 | 51.06 | activity |
| 70 | 1241 | 113 | 20 | 0.185 | 10 | 46 | 51.11 | 64.00 | 32.43 | core |
| 71 | 1245 | 15 | 3 | 0.091 | 4 | 8 | 66.67 | 62.67 | 53.41 | activity |
| 72 | 1246 | 9 | 4 | 0.109 | 4 | 12 | 100.00 | 61.33 | 55.42 | core |
| 73 | 1249 | 1 | 1 | 0.053 | 1 | 0 | 0.00 | 58.67 | 100.00 | activity |
| 74 | 1252 | 11 | 1 | 0.060 | 2 | 0 | 0.00 | 61.33 | 90.20 | activity |
| 75 | 8888 | 16 | 9 | 0.068 | 7 | 5 | 11.90 | 37.33 | 58.33 | activity |
| 76 | 9999 | 603 | 106 | 0.579 | 44 | 128 | 6.77 | 65.33 | 22.17 | core |

## Whole network

Whole network is composed of all members and their relationships who were include in a group. In this study, five indicators are used to measure whole network, including density, distance, centrality, cohesion subgroups, and structural hole. Their implications are as follows.

### Density

This indicator demonstrates the average strength of links which locate in the network actually. It is calculated by “the number of links included whole network in actually” (m) divided by “the number of links included whole network in theory” (n). In this study, the communications between parents and community childcare doctor are direct so that density equals to m/[n(n-1)]. The bigger density means that the strength of information delivery is stronger.

In this study, the density of intervention group (0.2912) is approximately five times of control group (0.0590), which indicates that the online-social-network-based parental health education intervention has a positive impact on enhancing parents’ knowledge, beliefs, and skills about child unintentional injuries. Additionally, due to that this study is conducted under the background of COVID-19, this study also provides a feasible evidence avoiding the obstacle of the offline social distance.

### Distance

In the whole network, distance demonstrates the length of the shortest path between two nodes in graph. There are two indicators are measured, including average distance and distanced-based cohesion (compactness). The calculation processes of distance are as follows.

First, distance matrix or generalized distance matrix should be listed. Generalized distance means the length of an optimum path. In whole network, totally n(n-1) pair actors are included. For average distance, this indicator is calculated by “the sum of distance matrix” divided by “the number of actors”. For distance-based cohesion (compactness), this indicator is calculated by the average of all multiplicative inverses determined based on each distance in the distance matrix, which demonstrates the degree of closeness between all social-network members.

In this study, the compactness of intervention group (21.6%) is approximately five times of control group (4.4%), which demonstrates that the online-social-network-based parental health education intervention can strength the communication between parents or parents and community childcare doctor. This intervention not only inspires parents’ study passion about child unintentional injuries, but also provides a convenient tool for parents which can let parents communicate with childcare doctor whenever and wherever. Compared traditional on-site intervention, online-social-network-based parental health education intervention provides a potential methods for community childcare doctor to implement community health management.

### Centrality

#### Degree centrality

This indicator demonstrates the number of nodes which links with parent A directly. For directed graph, degree centrality can be divided into in-degree centrality (the number of nodes which into the parent A) and out-degree centrality (the number of nodes which out the parent A). The bigger in-degree centrality means that parent A plays a role of studying child unintentional injuries. The bigger centrality means that parent A plays a role of teaching or communicating child unintentional injuries with other parents. The results are shown in Table S10~S11.

#### Betweenness centrality

If many geodesics are included between a pair of points (parent X and parent Z), among of them, some geodesics may pass point Y. The betweenness centrality of parent Y for parent X and parent Z means the odds of that “the number of geodesics through parent Y” divided by “the sum of geodesics between parent X and parent Z”. The bigger betweenness centrality means that parent Y has stronger ability to control the child-unintentional-injuries-related information delivery among other parents. The results are shown in Table S10~S11.

#### Closeness centrality

This indicator, which is calculated by distance, demonstrates the measurement of the degree to which the parents are not controlled by others. For parent A, it is calculated by the sum of its geodesics linking with others. The smaller closeness centrality means that parent A has stronger degree who is not controlled by others. The results are shown in Table S10~S11.

Table S16 The centrality of control group

| Number | ID | InDegree | NrmInDeg | OutDegree | NrmOutDeg | InCloseness | OutCloseness | Betweenness | nBetweenness |
| --- | --- | --- | --- | --- | --- | --- | --- | --- | --- |
| 1 | 1006 | 1.000 | 1.538 | 1.000 | 1.538 | 2.112 | 2.067 | 0.000 | 0.000 |
| 2 | 1012 | 0.000 | 0.000 | 0.000 | 0.000 | 1.515 | 1.515 | 0.000 | 0.000 |
| 3 | 1013 | 0.000 | 0.000 | 0.000 | 0.000 | 1.515 | 1.515 | 0.000 | 0.000 |
| 4 | 1016 | 0.000 | 0.000 | 0.000 | 0.000 | 1.515 | 1.515 | 0.000 | 0.000 |
| 5 | 1023 | 0.000 | 0.000 | 0.000 | 0.000 | 1.515 | 1.515 | 0.000 | 0.000 |
| 6 | 1025 | 0.000 | 0.000 | 0.000 | 0.000 | 1.515 | 1.515 | 0.000 | 0.000 |
| 7 | 1027 | 0.000 | 0.000 | 0.000 | 0.000 | 1.515 | 1.515 | 0.000 | 0.000 |
| 8 | 1029 | 0.000 | 0.000 | 0.000 | 0.000 | 1.515 | 1.515 | 0.000 | 0.000 |
| 9 | 1036 | 0.000 | 0.000 | 0.000 | 0.000 | 1.515 | 1.515 | 0.000 | 0.000 |
| 10 | 1038 | 0.000 | 0.000 | 0.000 | 0.000 | 1.515 | 1.515 | 0.000 | 0.000 |
| 11 | 1039 | 0.000 | 0.000 | 0.000 | 0.000 | 1.515 | 1.515 | 0.000 | 0.000 |
| 12 | 1040 | 0.000 | 0.000 | 1.000 | 1.538 | 1.515 | 2.110 | 0.000 | 0.000 |
| 13 | 1041 | 0.000 | 0.000 | 0.000 | 0.000 | 1.515 | 1.515 | 0.000 | 0.000 |
| 14 | 1049 | 0.000 | 0.000 | 0.000 | 0.000 | 1.515 | 1.515 | 0.000 | 0.000 |
| 15 | 1051 | 0.000 | 0.000 | 0.000 | 0.000 | 1.515 | 1.515 | 0.000 | 0.000 |
| 16 | 1055 | 0.000 | 0.000 | 0.000 | 0.000 | 1.515 | 1.515 | 0.000 | 0.000 |
| 17 | 1057 | 0.000 | 0.000 | 0.000 | 0.000 | 1.515 | 1.515 | 0.000 | 0.000 |
| 18 | 1059 | 0.000 | 0.000 | 0.000 | 0.000 | 1.515 | 1.515 | 0.000 | 0.000 |
| 19 | 1062 | 2.000 | 3.077 | 0.000 | 0.000 | 2.158 | 1.515 | 0.000 | 0.000 |
| 20 | 1065 | 0.000 | 0.000 | 0.000 | 0.000 | 1.515 | 1.515 | 0.000 | 0.000 |
| 21 | 1069 | 0.000 | 0.000 | 0.000 | 0.000 | 1.515 | 1.515 | 0.000 | 0.000 |
| 22 | 1078 | 0.000 | 0.000 | 0.000 | 0.000 | 1.515 | 1.515 | 0.000 | 0.000 |
| 23 | 1079 | 2.000 | 3.077 | 2.000 | 3.077 | 2.115 | 2.068 | 0.000 | 0.000 |
| 24 | 1083 | 0.000 | 0.000 | 0.000 | 0.000 | 1.515 | 1.515 | 0.000 | 0.000 |
| 25 | 1090 | 0.000 | 0.000 | 0.000 | 0.000 | 1.515 | 1.515 | 0.000 | 0.000 |
| 26 | 1093 | 1.000 | 1.538 | 1.000 | 1.538 | 2.094 | 2.058 | 0.000 | 0.000 |
| 27 | 1094 | 0.000 | 0.000 | 0.000 | 0.000 | 1.515 | 1.515 | 0.000 | 0.000 |
| 28 | 1096 | 0.000 | 0.000 | 0.000 | 0.000 | 1.515 | 1.515 | 0.000 | 0.000 |
| 29 | 1099 | 0.000 | 0.000 | 0.000 | 0.000 | 1.515 | 1.515 | 0.000 | 0.000 |
| 30 | 1101 | 0.000 | 0.000 | 0.000 | 0.000 | 1.515 | 1.515 | 0.000 | 0.000 |
| 31 | 1103 | 0.000 | 0.000 | 0.000 | 0.000 | 1.515 | 1.515 | 0.000 | 0.000 |
| 32 | 1104 | 6.000 | 9.231 | 5.000 | 7.692 | 2.119 | 2.071 | 26.750 | 0.643 |
| 33 | 1105 | 0.000 | 0.000 | 0.000 | 0.000 | 1.515 | 1.515 | 0.000 | 0.000 |
| 34 | 1106 | 1.000 | 1.538 | 1.000 | 1.538 | 2.138 | 1.515 | 0.000 | 0.000 |
| 35 | 1107 | 0.000 | 0.000 | 0.000 | 0.000 | 1.515 | 1.515 | 0.000 | 0.000 |
| 36 | 1108 | 2.000 | 3.077 | 2.000 | 3.077 | 2.105 | 2.063 | 1.250 | 0.030 |
| 37 | 1111 | 2.000 | 3.077 | 2.000 | 3.077 | 2.115 | 2.068 | 0.000 | 0.000 |
| 38 | 1123 | 0.000 | 0.000 | 0.000 | 0.000 | 1.515 | 1.515 | 0.000 | 0.000 |
| 39 | 1128 | 3.000 | 4.615 | 5.000 | 7.692 | 2.116 | 2.073 | 29.667 | 0.713 |
| 40 | 1130 | 0.000 | 0.000 | 0.000 | 0.000 | 1.515 | 1.515 | 0.000 | 0.000 |
| 41 | 1140 | 0.000 | 0.000 | 0.000 | 0.000 | 1.515 | 1.515 | 0.000 | 0.000 |
| 42 | 1143 | 0.000 | 0.000 | 0.000 | 0.000 | 1.515 | 1.515 | 0.000 | 0.000 |
| 43 | 1145 | 4.000 | 6.154 | 3.000 | 4.615 | 2.117 | 2.073 | 64.667 | 1.554 |
| 44 | 1151 | 0.000 | 0.000 | 0.000 | 0.000 | 1.515 | 1.515 | 0.000 | 0.000 |
| 45 | 1160 | 0.000 | 0.000 | 0.000 | 0.000 | 1.515 | 1.515 | 0.000 | 0.000 |
| 46 | 1167 | 0.000 | 0.000 | 0.000 | 0.000 | 1.515 | 1.515 | 0.000 | 0.000 |
| 47 | 1169 | 2.000 | 3.077 | 2.000 | 3.077 | 2.115 | 2.068 | 0.000 | 0.000 |
| 48 | 1170 | 0.000 | 0.000 | 0.000 | 0.000 | 1.515 | 1.515 | 0.000 | 0.000 |
| 49 | 1171 | 1.000 | 1.538 | 1.000 | 1.538 | 2.112 | 2.067 | 0.000 | 0.000 |
| 50 | 1173 | 0.000 | 0.000 | 0.000 | 0.000 | 1.515 | 1.515 | 0.000 | 0.000 |
| 51 | 1180 | 0.000 | 0.000 | 0.000 | 0.000 | 1.515 | 1.515 | 0.000 | 0.000 |
| 52 | 1190 | 0.000 | 0.000 | 0.000 | 0.000 | 1.515 | 1.515 | 0.000 | 0.000 |
| 53 | 1194 | 1.000 | 1.538 | 1.000 | 1.538 | 2.112 | 2.067 | 0.000 | 0.000 |
| 54 | 1198 | 0.000 | 0.000 | 0.000 | 0.000 | 1.515 | 1.515 | 0.000 | 0.000 |
| 55 | 1200 | 1.000 | 1.538 | 3.000 | 4.615 | 2.112 | 2.069 | 3.667 | 0.088 |
| 56 | 1201 | 2.000 | 3.077 | 2.000 | 3.077 | 2.114 | 2.069 | 11.000 | 0.264 |
| 57 | 1203 | 0.000 | 0.000 | 1.000 | 1.538 | 1.515 | 2.110 | 0.000 | 0.000 |
| 58 | 1212 | 0.000 | 0.000 | 0.000 | 0.000 | 1.515 | 1.515 | 0.000 | 0.000 |
| 59 | 1223 | 0.000 | 0.000 | 0.000 | 0.000 | 1.515 | 1.515 | 0.000 | 0.000 |
| 60 | 1224 | 2.000 | 3.077 | 4.000 | 6.154 | 2.106 | 2.069 | 53.500 | 1.286 |
| 61 | 1230 | 0.000 | 0.000 | 0.000 | 0.000 | 1.515 | 1.515 | 0.000 | 0.000 |
| 62 | 1248 | 1.000 | 1.538 | 1.000 | 1.538 | 2.112 | 2.067 | 0.000 | 0.000 |
| 63 | 1250 | 0.000 | 0.000 | 0.000 | 0.000 | 1.515 | 1.515 | 0.000 | 0.000 |
| 64 | 1257 | 0.000 | 0.000 | 1.000 | 1.538 | 1.515 | 2.110 | 0.000 | 0.000 |
| 65 | 8888 | 3.000 | 4.615 | 2.000 | 3.077 | 2.108 | 2.071 | 2.000 | 0.048 |
| 66 | 9999 | 16.000 | 24.615 | 13.000 | 20.000 | 2.125 | 2.079 | 256.500 | 6.166 |

Table S17 The centrality of intervention group

| Number | ID | InDegree | NrmInDeg | OutDegree | NrmOutDeg | InCloseness | OutCloseness | Betweenness | nBetweenness |
| --- | --- | --- | --- | --- | --- | --- | --- | --- | --- |
| 1 | 1003 | 1.000 | 1.333 | 2.000 | 2.667 | 3.420 | 3.539 | 1706.522 | 30.748 |
| 2 | 1004 | 9.000 | 12.000 | 13.000 | 17.333 | 3.505 | 3.634 | 156.994 | 2.829 |
| 3 | 1010 | 0.000 | 0.000 | 0.000 | 0.000 | 1.316 | 1.316 | 133.880 | 2.412 |
| 4 | 1011 | 0.000 | 0.000 | 0.000 | 0.000 | 1.316 | 1.316 | 122.561 | 2.208 |
| 5 | 1015 | 1.000 | 1.333 | 1.000 | 1.333 | 3.484 | 3.599 | 115.011 | 2.072 |
| 6 | 1017 | 2.000 | 2.667 | 2.000 | 2.667 | 3.492 | 3.609 | 112.525 | 2.027 |
| 7 | 1021 | 1.000 | 1.333 | 0.000 | 0.000 | 3.608 | 1.316 | 84.715 | 1.526 |
| 8 | 1022 | 2.000 | 2.667 | 3.000 | 4.000 | 3.434 | 3.609 | 51.990 | 0.937 |
| 9 | 1024 | 1.000 | 1.333 | 2.000 | 2.667 | 3.484 | 3.606 | 48.000 | 0.865 |
| 10 | 1031 | 0.000 | 0.000 | 0.000 | 0.000 | 1.316 | 1.316 | 36.367 | 0.655 |
| 11 | 1033 | 0.000 | 0.000 | 0.000 | 0.000 | 1.316 | 1.316 | 35.408 | 0.638 |
| 12 | 1034 | 0.000 | 0.000 | 0.000 | 0.000 | 1.316 | 1.316 | 24.196 | 0.436 |
| 13 | 1035 | 0.000 | 0.000 | 0.000 | 0.000 | 1.316 | 1.316 | 18.010 | 0.324 |
| 14 | 1037 | 0.000 | 0.000 | 0.000 | 0.000 | 1.316 | 1.316 | 14.805 | 0.267 |
| 15 | 1042 | 0.000 | 0.000 | 0.000 | 0.000 | 1.316 | 1.316 | 14.311 | 0.258 |
| 16 | 1044 | 2.000 | 2.667 | 2.000 | 2.667 | 3.488 | 3.608 | 10.960 | 0.197 |
| 17 | 1045 | 0.000 | 0.000 | 0.000 | 0.000 | 1.316 | 1.316 | 9.247 | 0.167 |
| 18 | 1046 | 4.000 | 5.333 | 3.000 | 4.000 | 3.447 | 3.608 | 8.076 | 0.146 |
| 19 | 1054 | 1.000 | 1.333 | 1.000 | 1.333 | 3.484 | 3.599 | 7.933 | 0.143 |
| 20 | 1056 | 9.000 | 12.000 | 7.000 | 9.333 | 3.501 | 3.616 | 6.535 | 0.118 |
| 21 | 1058 | 9.000 | 12.000 | 6.000 | 8.000 | 3.503 | 3.613 | 6.223 | 0.112 |
| 22 | 1060 | 0.000 | 0.000 | 0.000 | 0.000 | 1.316 | 1.316 | 5.500 | 0.099 |
| 23 | 1063 | 1.000 | 1.333 | 1.000 | 1.333 | 3.484 | 3.599 | 4.550 | 0.082 |
| 24 | 1072 | 0.000 | 0.000 | 0.000 | 0.000 | 1.316 | 1.316 | 3.750 | 0.068 |
| 25 | 1074 | 16.000 | 21.333 | 15.000 | 20.000 | 3.515 | 3.634 | 2.739 | 0.049 |
| 26 | 1075 | 0.000 | 0.000 | 0.000 | 0.000 | 1.316 | 1.316 | 2.345 | 0.042 |
| 27 | 1077 | 1.000 | 1.333 | 1.000 | 1.333 | 3.484 | 3.599 | 1.397 | 0.025 |
| 28 | 1080 | 9.000 | 12.000 | 5.000 | 6.667 | 3.501 | 3.613 | 0.250 | 0.005 |
| 29 | 1085 | 3.000 | 4.000 | 2.000 | 2.667 | 3.487 | 3.601 | 0.200 | 0.004 |
| 30 | 1087 | 0.000 | 0.000 | 0.000 | 0.000 | 1.316 | 1.316 | 0.000 | 0.000 |
| 31 | 1089 | 0.000 | 0.000 | 0.000 | 0.000 | 1.316 | 1.316 | 0.000 | 0.000 |
| 32 | 1091 | 0.000 | 0.000 | 0.000 | 0.000 | 1.316 | 1.316 | 0.000 | 0.000 |
| 33 | 1095 | 4.000 | 5.333 | 3.000 | 4.000 | 3.490 | 3.608 | 0.000 | 0.000 |
| 34 | 1100 | 2.000 | 2.667 | 0.000 | 0.000 | 3.549 | 1.316 | 0.000 | 0.000 |
| 35 | 1102 | 0.000 | 0.000 | 0.000 | 0.000 | 1.316 | 1.316 | 0.000 | 0.000 |
| 36 | 1109 | 4.000 | 5.333 | 8.000 | 10.667 | 3.492 | 3.620 | 0.000 | 0.000 |
| 37 | 1113 | 1.000 | 1.333 | 1.000 | 1.333 | 3.484 | 3.599 | 0.000 | 0.000 |
| 38 | 1116 | 2.000 | 2.667 | 2.000 | 2.667 | 3.485 | 3.602 | 0.000 | 0.000 |
| 39 | 1119 | 15.000 | 20.000 | 16.000 | 21.333 | 3.513 | 3.637 | 0.000 | 0.000 |
| 40 | 1129 | 1.000 | 1.333 | 1.000 | 1.333 | 3.484 | 3.599 | 0.000 | 0.000 |
| 41 | 1132 | 5.000 | 6.667 | 3.000 | 4.000 | 3.497 | 3.609 | 0.000 | 0.000 |
| 42 | 1136 | 2.000 | 2.667 | 1.000 | 1.333 | 3.485 | 3.599 | 0.000 | 0.000 |
| 43 | 1139 | 0.000 | 0.000 | 0.000 | 0.000 | 1.316 | 1.316 | 0.000 | 0.000 |
| 44 | 1146 | 8.000 | 10.667 | 9.000 | 12.000 | 3.501 | 3.620 | 0.000 | 0.000 |
| 45 | 1147 | 3.000 | 4.000 | 2.000 | 2.667 | 3.487 | 3.601 | 0.000 | 0.000 |
| 46 | 1149 | 1.000 | 1.333 | 1.000 | 1.333 | 3.484 | 3.599 | 0.000 | 0.000 |
| 47 | 1154 | 1.000 | 1.333 | 1.000 | 1.333 | 3.484 | 3.599 | 0.000 | 0.000 |
| 48 | 1157 | 0.000 | 0.000 | 0.000 | 0.000 | 1.316 | 1.316 | 0.000 | 0.000 |
| 49 | 1161 | 2.000 | 2.667 | 2.000 | 2.667 | 3.485 | 3.601 | 0.000 | 0.000 |
| 50 | 1174 | 7.000 | 9.333 | 5.000 | 6.667 | 3.466 | 3.575 | 0.000 | 0.000 |
| 51 | 1189 | 7.000 | 9.333 | 8.000 | 10.667 | 3.495 | 3.618 | 0.000 | 0.000 |
| 52 | 1191 | 1.000 | 1.333 | 1.000 | 1.333 | 3.380 | 3.501 | 0.000 | 0.000 |
| 53 | 1193 | 2.000 | 2.667 | 1.000 | 1.333 | 3.492 | 3.599 | 0.000 | 0.000 |
| 54 | 1196 | 11.000 | 14.667 | 15.000 | 20.000 | 3.506 | 3.637 | 0.000 | 0.000 |
| 55 | 1197 | 0.000 | 0.000 | 0.000 | 0.000 | 1.316 | 1.316 | 0.000 | 0.000 |
| 56 | 1199 | 0.000 | 0.000 | 0.000 | 0.000 | 1.316 | 1.316 | 0.000 | 0.000 |
| 57 | 1202 | 4.000 | 5.333 | 4.000 | 5.333 | 3.493 | 3.606 | 0.000 | 0.000 |
| 58 | 1204 | 1.000 | 1.333 | 1.000 | 1.333 | 3.426 | 3.538 | 0.000 | 0.000 |
| 59 | 1209 | 0.000 | 0.000 | 0.000 | 0.000 | 1.316 | 1.316 | 0.000 | 0.000 |
| 60 | 1215 | 1.000 | 1.333 | 1.000 | 1.333 | 3.484 | 3.599 | 0.000 | 0.000 |
| 61 | 1218 | 3.000 | 4.000 | 7.000 | 9.333 | 3.488 | 3.618 | 0.000 | 0.000 |
| 62 | 1220 | 0.000 | 0.000 | 0.000 | 0.000 | 1.316 | 1.316 | 0.000 | 0.000 |
| 63 | 1222 | 7.000 | 9.333 | 9.000 | 12.000 | 3.501 | 3.625 | 0.000 | 0.000 |
| 64 | 1225 | 1.000 | 1.333 | 0.000 | 0.000 | 3.463 | 1.316 | 0.000 | 0.000 |
| 65 | 1226 | 0.000 | 0.000 | 0.000 | 0.000 | 1.316 | 1.316 | 0.000 | 0.000 |
| 66 | 1227 | 0.000 | 0.000 | 0.000 | 0.000 | 1.316 | 1.316 | 0.000 | 0.000 |
| 67 | 1229 | 1.000 | 1.333 | 1.000 | 1.333 | 3.484 | 3.599 | 0.000 | 0.000 |
| 68 | 1234 | 0.000 | 0.000 | 1.000 | 1.333 | 1.316 | 3.731 | 0.000 | 0.000 |
| 69 | 1239 | 3.000 | 4.000 | 4.000 | 5.333 | 3.493 | 3.611 | 0.000 | 0.000 |
| 70 | 1241 | 9.000 | 12.000 | 8.000 | 10.667 | 3.503 | 3.621 | 0.000 | 0.000 |
| 71 | 1245 | 4.000 | 5.333 | 3.000 | 4.000 | 3.495 | 3.609 | 0.000 | 0.000 |
| 72 | 1246 | 4.000 | 5.333 | 4.000 | 5.333 | 3.493 | 3.609 | 0.000 | 0.000 |
| 73 | 1249 | 0.000 | 0.000 | 1.000 | 1.333 | 1.316 | 3.731 | 0.000 | 0.000 |
| 74 | 1252 | 2.000 | 2.667 | 2.000 | 2.667 | 3.490 | 3.606 | 0.000 | 0.000 |
| 75 | 8888 | 5.000 | 6.667 | 7.000 | 9.333 | 3.453 | 3.582 | 0.000 | 0.000 |
| 76 | 9999 | 43.000 | 57.333 | 40.000 | 53.333 | 3.561 | 3.684 | 0.000 | 0.000 |

### Cohesion subgroups

Cohesion subgroups meet one of the below requirements, such as relatively strong, direct, closely, usual, or active relationships. There are four aspects used to explain cohesion subgroups: (1) relationship reciprocity, (2) proximity or accessibility among subgroup members, (3) frequency of relationships among members within a subgroup (degree of points), and and (4) density of relationships among members of a subgroup relative to the relationship between internal and external members. Due to child-unintentional-injuries-related information is delivered in WeChat groups and parents communicate with others mutually, relationship reciprocity is selected to analyze cohesion subgroups including components and cliques.

#### Components

A point set is said to be a component if any two points of the set can be connected by a certain way. For directed graph, it is strong components. For undirected graph, it is weak components.

#### Cliques

Cliques are a maximal complete sub-graph including three nodes at least. For directed graph, based on the definition of cliques, only reciprocal relationships can be included. In addition, all cliques can be simplified, according to clique-by-clique co-membership matrix. Cliques with repeat members can be simplified to a single group, and cliques without repeat members remain the same.

### Structural hole

Structural holes were first suggested by Burt, and were used in this study to identify non-redundant relationships between two parents. Supplementary Material Figure 4 provides details regarding the redundant and non-redundant relationships in the network. Figure S4a) shows a structural hole in which ego has an association not only with Parent A, but also with Parent B, while Parent A and Parent B have no relationship; this represents a hole because ego must deliver information to parent A and parent B, respectively. In Figure S4b), a structural hole is not present because Parent A has an association with Parent B, and ego only needs to deliver information to Parent A, who can then provide the information to Parent B; thus, the relationship between ego and Parent B is redundant.

There are four important indicators for structural holes: (1) the effective size of the network: this indicator represents the number of non-redundant factors in Parent A’s network; (2) efficiency: for Parent A, this indicator represents the ratio of effective size to actual size; (3) constraint: this demonstrates the impact of the presence of structural holes in an ego network; and (4) hierarchy: this demonstrates the extent to which constraint focuses on each parent and the community childcare doctor. Among these, the effective size of the network and constraint are very important.


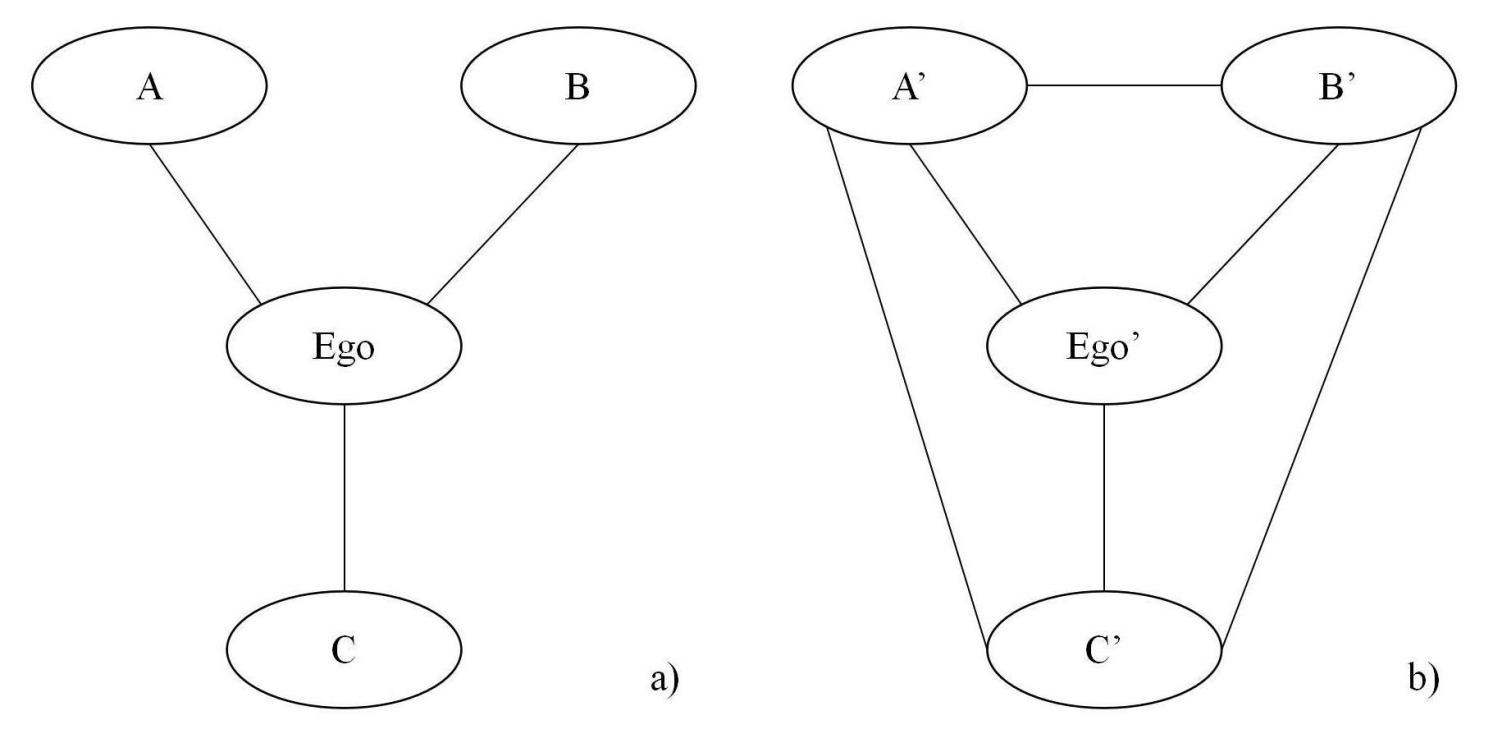


Supplementary Material Figure S4 The example of structural holes

In this study, effective size and constraint are selected to reflect structural hole.
